# Recognition of AMP, ADP and ATP through Cooperative Binding by Cu(II) and Zn(II) Complexes Containing Urea and/or Phenylboronic Acid Moieties

**DOI:** 10.3390/molecules23020479

**Published:** 2018-02-22

**Authors:** Israel Carreira-Barral, Isabel Fernández-Pérez, Marta Mato-Iglesias, Andrés de Blas, Carlos Platas-Iglesias, David Esteban-Gómez

**Affiliations:** 1 Departamento de Química, Facultade de Ciencias, Universidade da Coruña, Centro de Investigacións Científicas Avanzadas (CICA), 15071 A Coruña, Galicia, Spain; icarreira@ubu.es (I.C.-B.); sabela@udc.es (I.F.-P.); mmato@udc.es (M.M.-I.); andres.blas@udc.es (A.B.); carlos.platas.iglesias@udc.es (C.P.-I.); 2 Departamento de Química, Facultad de Ciencias, Universidad de Burgos, 09001 Burgos, Spain

**Keywords:** molecular sensors, metal complexes, cooperative binding, multitopic receptor, hydrogen bonding, urea group, phenylboronic moiety

## Abstract

We report a series of Cu(II) and Zn(II) complexes with different ligands containing a dipicolyl unit functionalized with urea groups that may contain or not a phenylboronic acid function. These complexes were designed for the recognition of phosphorylated anions through coordination to the metal ion reinforced by hydrogen bonds involving the anion and NH groups of urea. The complexes were isolated and several adducts with pyrophosphate were characterized using X-ray diffraction measurements. Coordination of one of the urea nitrogen atoms to the metal ion promoted the hydrolysis of the ligands containing 1,3-diphenylurea units, while ligands bearing 1-ethyl-3-phenylurea groups did not hydrolyze significantly at room temperature. Spectrophotometric titrations, combined with ^1^H and ^31^P NMR studies, were used in investigating the binding of phosphate, pyrophosphate (PPi), and nucleoside 5′-polyphosphates (AMP, ADP, ATP, CMP, and UMP). The association constants determined in aqueous solution (pH 7.0, 0.1 M MOPS) point to a stronger association with PPi, ADP, and ATP as compared with the anions containing a single phosphate unit. The [CuL^4^]^2+^ complex shows important selectivity for pyrophosphate (PPi) over ADP and ATP.

## 1. Introduction

Phosphorylated metabolites are involved in biologically relevant processes [1], such as transport of chemical energy [2] (adenosine triphosphate, ATP, and pyrophosphate (PPi), signal transduction and transmission [3,4,5], activation of platelets [6], and protein kinases [7] (adenosine monophosphate, AMP, and adenosine diphosphate (ADP), or DNA replication catalyzed by DNA polymerase, a process in which PPi plays an important role [8,9,10]. Thus, the design and synthesis of artificial receptors that bind phosphorylated anions is an important scientific goal with potential application in sensing, optical imaging and in vitro clinical diagnosis [11,12,13,14,15,16,17,18,19]. For instance, pyrophosphate (PPi) is one of the hydrolysis products of ATP and is released in DNA/RNA polymerase reactions [20,21]. Abnormal levels of this anion have been related to a number of diseases, including arthritis and cancer [22].

The interaction of Cu(II) and Zn(II) ions with nucleoside 5′-polyphosphates (AMP, ADP, ATP, CMP, and UMP) were studied in detail by Sigel et al. in the early 90’s. [23,24,25,26,27] The formation of macrochelates was clearly defined for adenine nucleotides, thanks to the coordination of both the phosphate chains and the nitrogen N-7 of the purine residue. [28,29] Since then, different types of molecular receptors have been used for the recognition of phosphorylated anions in aqueous media [1]: (1) Linear or macrocyclic positively charged receptors, generally polyammonium systems [30,31,32,33] or receptors containing guanidinium or imidazolium groups [34,35]; (2) Metal complexes, generally coordinatively unsaturated Cu^2+^ or Zn^2+^ complexes, with acyclic (i.e., dipicolyl or terpyridyl derivatives [36,37,38,39,40,41,42] or Schiff base ligands [43]) or macrocyclic ligands [44,45,46]; (3) Ditopic receptors that combine a metal coordinating unit and hydrogen-bond donors to reinforce anion binding [47,48].

In a previous work, we investigated the ability of the Cu^2+^ and Zn^2+^ complexes derived from **L^2^** to bind anions through cooperative binding, involving (i) coordination of the anion to the metal ions, and (ii) hydrogen-bonding interactions involving the coordinated anion and the NH groups of the urea moiety as hydrogen-bond donors [49]. More recently we have shown that the [CuL^2^]^2+^ complex binds sulphate through such cooperative binding [50]. Thus, we envisaged that the [CuL^2^]^2+^ complex and related derivatives could be useful for the recognition of phosphorylated anions. Herein, we report a series of ligands containing a bis(pyridin-2-ylmethyl)amine unit for coordination to Cu^2+^ and Zn^2+^, and a urea moiety that is expected to reinforce anion binding through hydrogen-bonding interactions. The flexibility of the receptor was modulated by including a benzyl (**L^1^** and **L^2^**) or ethyl (**L^3^** and **L^4^**) spacer connecting the urea and bis(pyridin-2-ylmethyl)amine units. When considering the ability of phenylboronic acids to bind *cis*-diol groups [51,52,53], we also prepared ligands **L^1^**, **L^3^**, and **L^5^** in an attempt to reinforce the binding to AMP, ADP, and ATP. We report a series of X-ray structures and ^1^H and ^31^P NMR studies that provide insight into the nature of the interactions established between the Zn^2+^ and Cu^2+^ complexes and different anions. Spectrophotometric titrations were used to determine the stoichiometries and association constants characterizing anion binding.

## 2. Results

### 2.1. Synthesis and Characterization of the Ligands and Metal Complexes

**Scheme 1:**
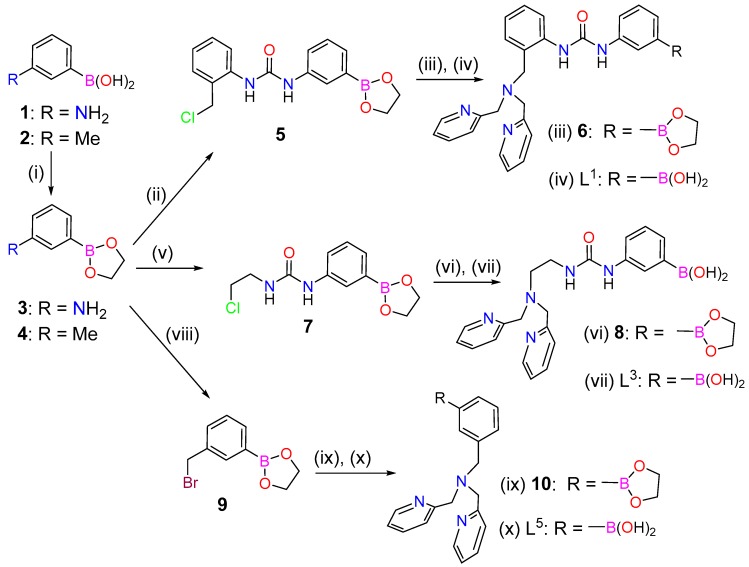
Synthesis of ligands **L^1^**, **L^3^** and **L^5^**: (i) Ethane-1,2-diol, Δ, 100% (**3**), 85% (**4**); (ii) Compound **3**, 1-(Chloromethyl)-2-isocyanatobenzene, Et_2_O, r.t., 16 h, 91%; (iii) Bis(pyridin-2-ylmethyl)amine, CH_3_CN, DIPEA, 70 °C, 5 d; (iv) MPLC, neutral Al_2_O_3_, CH_2_Cl_2_:MeOH, 40% (two steps); (v) Compound **3**, 1-Chloro-2-isocyanatoethane, Et_2_O, r.t., 16 h, 89%; (vi) Bis(pyridin-2-ylmethyl)amine, CH_3_CN, DIPEA, 70 °C, 5 d; (vii) MPLC, neutral Al_2_O_3_, CH_2_Cl_2_:MeOH, 24% (two steps); (viii) Compound **4**, *N*-bromosuccinimide (NBS), dibenzoylperoxide, CCl_4_, Δ, 16 h, 54%; (ix) Bis(pyridin-2-ylmethyl)amine, CH_3_CN, DIPEA, Δ, 16 h; (x) MPLC, neutral Al_2_O_3_, CH_2_Cl_2_:MeOH, 42% (two steps).

The preparation of **L^2^** and the corresponding Cu^2+^ and Zn^2+^ complexes using sulphate and nitrate as counterions were described in a previous work [49]. The syntheses of **L^1^** and **L^3^** started from (3-aminophenyl)boronic acid (**1**), which was protected using ethane-1,2-diol to give compound **3** in quantitative yield (Scheme 1). Compound **3** was subsequently reacted with 1-(chloromethyl)-2-isocyanatobenzene or 1-chloro-2-isocyanatoethane to yield compounds **5** and **7**, respectively. Alkylation of bis(pyridin-2-ylmethyl)amine with **5** (or **7**) provided compound **6** (or **8**), which were purified using MPLC on neutral Al_2_O_3_. This afforded directly ligands **L^1^** and **L^3^**, thanks to the deprotection of the phenylboronic acid group during the chromatographic separation. The alkylation of bis(pyridin-2-ylmethyl)amine with **5** and **7** were found to proceed in rather low yields (Scheme 1).

The synthesis of **L^5^** started from *m*-tolylboronic acid (**2**), which was converted to the bromomethyl derivative **9** following the literature procedure [53]. Alkylation of bis(pyridin-2-ylmethyl)amine with compound **9** in acetonitrile using *N,N*-Diisopropylethylamine (DIPEA) as a base and subsequent purification gave **L^5^**.

**Scheme 2:**
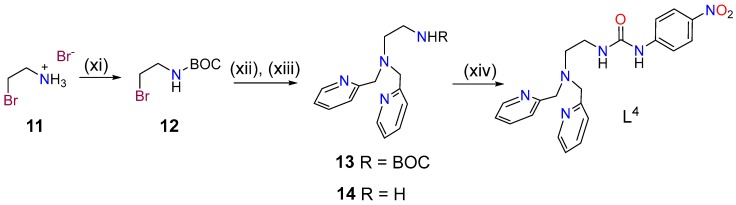
Synthesis of ligand **L^4^**: (xi) di-*tert*-butyl dicarbonate, dioxane, 0 °C, 48 h, 80% (**12**); (xii) Bis(pyridin-2-ylmethyl)amine, CH_3_CN, DIPEA, 70 °C, 3 d, 39%; (xiii) trifluoroacetic acid (TFA:CH_2_Cl_2_ (1:1), r.t., 16 h, 96%; (xiv) 1-isocyanato-4-nitrobenzene, CHCl_3_, r.t., 2 h, 85%.

The synthesis of ligand L^4^ was initiated by protecting compound **11** with di-*tert*-butyl dicarbonate and subsequent reaction of **12** with bis(pyridin-2-ylmethyl)amine in acetonitrile using DIPEA as a base (Scheme 2). Deprotection of compound **13** with trifluoroacetic acid (TFA) was followed by reaction of **14** with 1-isocyanato-4-nitrobenzene affording ligand L^4^.

The reaction of 1 equivalent of ligand (L^n^; (*n* = 1, 3, 4 or 5)) with 1 equiv of hydrated M(ClO_4_)_2_ salts (M = Cu or Zn) in THF (**L^1^**), acetonitrile (**L^3^** and **L^5^**), or methanol (**L^4^**) at room temperature, provided the desired complexes as the perchlorate salts (except for [ZnL^5^](ClO_4_)_2_ that was prepared in situ for ^1^H-NMR measurements). In the case of **L^4^**, the [CuL^4^(SO_4_)] complex was also prepared by a similar procedure in methanol. The complexes were isolated in 82–97% yields, with the exception of those derived from **L^3^**, which were obtained in lower yields (49% and 59% for [ZnL^3^](ClO_4_)_2_ and [CuL^3^](ClO_4_)_2_, respectively).

As expected, the IR spectra (KBr discs) feature bands due to ν(N-H) and ν(C=O) modes associated to the presence of the urea group in **L^1^**, **L^3^**, and **L^4^** derivatives, while ν(O-H) and ν(O-B-O) modes are also observed when the boronic moiety is present (**L^1^**, **L^3^**, and **L^5^**). In all cases, the bands corresponding to the ν(Ph/Py) mode are present, and the coordination of the metal ions induces significant shifts with respect to the corresponding free ligands (see Materials and Methods Section). It is noticeable that the bands corresponding to the ν_as_(Cl-O) (stretching) and δ_as_(O-Cl-O) (bending) modes of the perchlorate groups appear without splitting for all complexes at ca. 1100 and 625 cm^−1^, respectively, as befit uncoordinated anions [54,55]. In the case of [CuL^4^(SO_4_)], the bands corresponding to the ν_a_(S-O) and ν_a_(O-S-O) modes are splitted, as expected for a coordinated sulphate anion with low symmetry. This is in agreement with UV-vis diffuse reflectance spectroscopy, which provided different positions for the *d*-*d* absorption band observed for [CuL^4^(SO_4_)] and [CuL^4^](ClO_4_)_2_ (706 and 633 nm, respectively).

Conductivity measurements were carried out in 10^−3^ M methanol solutions at 25 °C. The molar conductivity of the perchlorate salts (154.9–171.3 cm^2^ Ω^−1^ mol^−1^) are in the range reported for 2:1 electrolytes [56], indicating that these complexes are completely dissociated in methanol solution. In the case of [CuL^4^(SO_4_)], the low solubility of this charge-neutral complex prevented us from determining its molar conductivity value, confirming that the sulphate anion is directly bonded to the Cu^2+^ ion.

### 2.2. X-ray Crystal Structures

**Figure 1:**
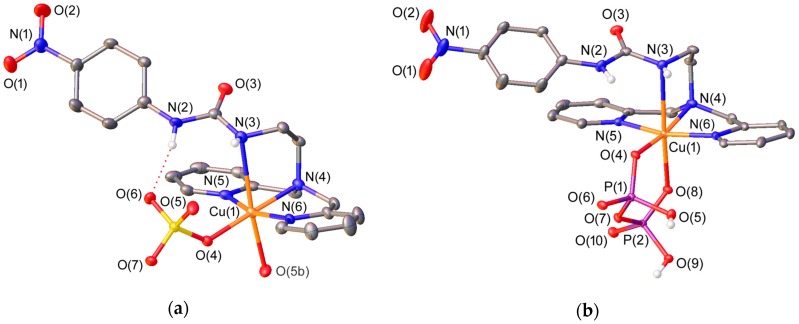
(**a**) X-ray crystal structure of [CuL^4^(SO_4_)] with labelling scheme; (**b**) X-ray crystal structure of [CuL^4^(H_2_PPi)] with labelling scheme. The Oak Ridge Thermal-Ellipsoid Plot Program (ORTEP) plots are at the 30% probability level. Symmetry transformations used to generate equivalent atoms for [CuL^4^(SO_4_)]: #2 −x + 1, y − 1/2,−z + 3/2.

**Figure 2:**
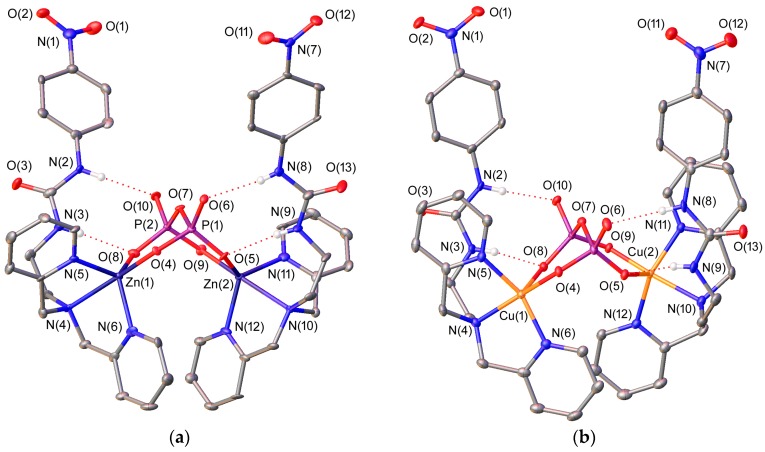
(**a**) X-ray crystal structure of [{ZnL^4^}_2_(μ-PPi)] with labelling scheme; (**b**) X-ray crystal structure of [{CuL^4^}_2_(μ-PPi)] with labelling scheme. The ORTEP plots are at the 30% probability level.

The solid state structures of different complexes of **L^4^** were investigated by using X-ray diffraction measurements. Table 1 presents the bond distances of the metal coordination environments, while views of the structures are shown in Figure 1 and Figure 2. The complexes differ in the nature of the metal ion (Zn^2+^ or Cu^2+^) and the anion (sulphate or pyrophosphate). The Cu^2+^ ions in [CuL^4^(SO_4_)] and [CuL^4^(H_2_PPi)] complexes present octahedral coordination with a strong Jahn-Teller distortion [57]. The ligand adopts an ‘open wing butterfly’ conformation that is similar to those observed for the Cu^2+^ complexes of L^2^ [49]. The equatorial plane of the coordination polyhedron in [CuL^4^(SO_4_)] is defined by the donor atoms of the dipicolylamine unit [N(4), N(5) and N(6)] and an oxygen atom of the anion [O(4)] (mean deviation from planarity: 0.04 Å), with the metal ion being placed 0.01 Å below this plane in [CuL^4^(SO_4_)] and 0.12 Å above it in [CuL^4^(H_2_PPi)]. The apical positions in [CuL^4^(SO_4_)] are occupied by one of the nitrogen atoms of the urea subunit [N(3)] and an oxygen atom of a sulphate anion of an adjacent complex molecule [O(5b)] (Figure 1). Thus, the presence of μ_2_–η^1^:η^1^-sulphate anions bridging two neighboring complex molecules leads to the formation of a coordination polymer (Figure S1, Supplementary Materials). In [CuL^4^(H_2_PPi)], the apical positions are delineated by one of the nitrogen atoms of the urea subunit [N(3)] and an oxygen atom of the H_2_PPi^2−^ anion [O(8)], which coordinates in a κ^2^-H_2_PPi^2−^ mode forming a six-membered chelate ring.

The *cis* angles of the coordination polyhedron (81.0°–98.8° in [CuL^4^(SO_4_)] and 82.4°–98.4° in [CuL^4^(H_2_PPi)]) are relatively close to the one expected for a regular octahedral coordination (90°), whereas the corresponding *trans* angles (162.7°–178.0° for [CuL^4^(SO_4_)] and 164.4–173.8 for [CuL^4^(H_2_PPi)]) deviate up to 18° from the ideal one (180°) (Figure S2, Supplementary Materials).

The sulphate anion in [CuL^4^(SO_4_)] interacts with the metal ion via a coordinate bond and with the urea moiety through a moderate hydrogen-bonding interaction, the latter involving one of the NH groups of the urea unit as hydrogen-bond donor and an oxygen atom of the anion as hydrogen-bond acceptor [N(2)⋯O(6) 2.768(3) Å; N(2)-H(2N)⋯O(6), 1.97(3) Å; N(2)-H(2N)⋯O(6) 161(3)°]. The value of the S(1)-O(6)-H(2N) angle (116.8°) is close to the ideal one (122° ± 10°) [58]. The interaction of the anion with another complex molecule causes sulphate to move away from the N(3)-H(3N) fragment, which interacts with an oxygen atom of another sulphate anion via an intermolecular hydrogen-bonding interaction.

We have also obtained single crystals suitable for X-ray diffraction measurements of [{ZnL^4^}_2_(μ-PPi)] and [{CuL^4^}_2_(μ-PPi)] (Figure 2), in which the fully deprotonated pyrophosphate anion acts as a bridging μ_2_κ^4^-PPi^4−^ ligand forming two six-membered chelate rings [59,60]. This bridging mode of the anion has been previously reported by Hong and coworkers in the solid state for dinuclear Zn^2+^ complexes derived from di(2-picolyl)amine [61,62]. The ligand in [{CuL^4^}_2_(μ-PPi)] adopts an ′open wing butterfly′ conformation (Figure 2), which results in a square pyramidal geometry around the Cu^2+^ ion. The basal plane is defined by the three donor atoms of the dipicolylamine unit [N(4), N(5), and N(6) for Cu(1) and N(10), N(11) and N(12) for Cu(2)] and an oxygen atom of pyrophosphate [O(4) or O(9)] [mean deviation from planarity: 0.03 Å for Cu(1) and 0.05 Å for Cu(2)], while the apical position is occupied by another oxygen atom of the anion [O(8) or O(5)]. The metal ion is placed 0.29 Å [Cu(1)] or 0.28 Å [Cu(2)] above the basal plane. The *cis* angles of this plane (82.8°–95.7°) and those involving the oxygen atom occupying the apical position, the metal centre and the donor atoms of the equatorial plane (93.3°–102.5°), as well as the *trans* angles (157.4°–166.8°), are relatively close to the ideal ones (90 and 180°, respectively). The index of trigonality τ, which amounts to 0.12 for Cu(1) and 0.16 for Cu(2) (τ = 0 for an ideal square pyramidal geometry and τ = 1 for a regular trigonal bipyramidal environment) [63] is in agreement with the square pyramidal polyhedron.

The ligand **L^4^** in [{ZnL^4^}_2_(μ-PPi)] adopts a ′bent propeller′ conformation and wraps around the metal ions providing trigonal bipyramidal coordination environments. The equatorial plane of the bipyramid is defined by the nitrogen atoms of the pyridine units [N(5) and N(6) for Zn(1) and N(11) and N(12) for Zn(2)] and an oxygen atom of the pyrophosphate anion [O(8) for Zn(1) and O(5) for Zn(2)]. The apical positions are occupied by the amine nitrogen atom and one of the oxygen atoms of the anion [N(4) and O(4) for Zn(1) and N(10) and O(9) for Zn(2)]. The Zn^2+^ ion is placed 0.24 Å [Zn(1)] or 0.25 Å [Zn(2)] above the equatorial plane. The angles involving the donor atoms of this plane (113.9°–124.7°), as well as the *trans* angle (170.4°–170.6°) are close to the ones expected for a regular trigonal bipyramid (120° and 180°, respectively). The index of trigonality τ amounts to 0.76 for both metals [63], which is in agreement with the indicated geometry.

The PPi^4−^ anions in both [{ZnL^4^}_2_(μ-PPi)] and [{CuL^4^}_2_(μ-PPi)] show a cooperative binding in which the anion interacts simultaneously with the metal ion via coordinate bonds, and with the urea moiety through Y-shape hydrogen-bonding interactions. The N-H(N)-O angles and distances (Table 2) show that these interactions are rather symmetrical and can be regarded as moderate, although the N⋯O distances point to a slightly weaker interaction in the Zn^2+^ complex. In all cases, the P-O-H(N) angles (109°–122°) are close to the ideal value for a tetrahedral anion (122° ± 12°) [58].

### 2.3. Stability of the Complexes Toward Hydrolysis

**Scheme 3:**
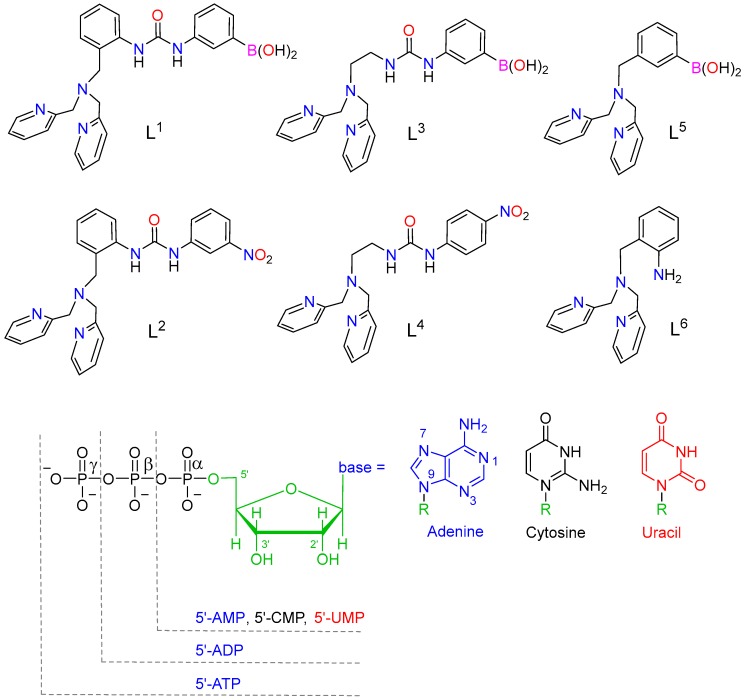
Ligands and phosphorylated anions discussed in the present work.

**Figure 3:**
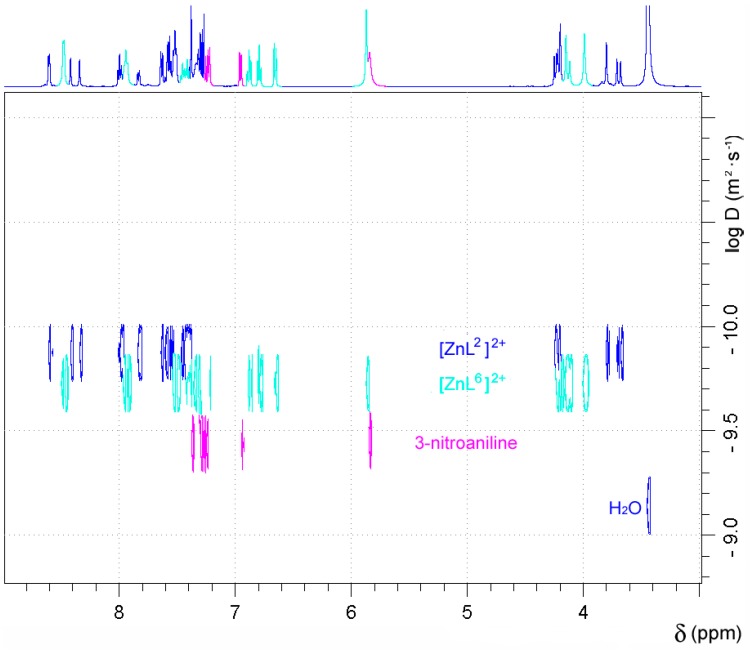
DOSY ^1^H-NMR spectrum (500 MHz, 298 K) recorded for [ZnL^2^]^2+^ 168 h after dissolution of the complex in dmso-*d*_6_.

The ^1^H-NMR spectra of the Zn^2+^ complexes of both **L^1^** and **L^2^** recorded in wet dmso-*d*_6_ were observed to change over time, which caused an increase of the number and complexity of the signals (Figures S3 and S4, Supplementary Materials). This suggests that both [ZnL^1^]^2+^ and [ZnL^2^]^2+^ complexes hydrolyse in aqueous solution to form the [ZnL^6^]^2+^ complex (Scheme 3), 2-aminophenylboronic acid or 3-nitroaniline, and carbon dioxide. The presence of a 3-nitroaniline molecule in the molecular structure of [CuL^2^(μ-SO_4_)]_2_ reported recently also points in this direction [50]. In the case of [ZnL^2^](ClO_4_)_2_, new resonances appear in the ^1^H-NMR spectrum from 7.35 to 6.60 ppm, none of which correspond to the complex or the free ligand. The presence of a signal at 6.94 ppm due to 3-nitroaniline confirmed the hydrolysis of the complex. The relative intensity of the signals attributable to the [ZnL^2^]^2+^ species and 3-nitroaniline indicate that 6% of the complex has hydrolysed after four hours at room temperature, while after 48, 96, and 168 h this percentage increases to 28%, 43%, and 49%, respectively. A similar situation was observed for [ZnL^1^]^2+^, which hydrolysed to a slightly lower extent after 4 h (~5%). ^1^H DOSY experiments performed for [ZnL^2^]^2+^ 168 h after dissolution of the complex allowed discriminating the three species that are present in solution on the basis of their different diffusion coefficients (Figure 3). Taking the diffusion coefficient of dmso-*d*_5_ as a reference (*D* = 6.81 × 10^−10^ m^2^·s^−1^) [64], these studies provided diffusion coefficients *D* at 25 °C of 1.34 × 10^−10^ m^2^·s^−1^, 1.85 × 10^−10^ m^2^·s^−1^, and 3.64 × 10^−10^ m^2^·s^−1^ for [ZnL^2^]^2+^, [ZnL^6^]^2+^, and 3-nitroaniline, respectively.

The hydrolysis of the urea unit in [CuL^2^]^2+^ was monitored in aqueous solution by following the absorption band of 3-nitroaniline (λ_max_ = 357 nm, ε = 1486 M^−1^·cm^−1^) over time and employing the method of initial rates (Figure S5, Supplementary Materials). The observed pseudo-first-order rate constants (*k*_obs_) and half-life times (*t*_1/2_) were determined by fitting the data obtained at 390 nm using two temperatures (25 and 50 °C, *I* = 0.1 M NaClO_4_) and two pH values (5.5 and 7.0). In line with previous studies [65], our results did not reveal significant pH dependence of the *k*_obs_ values in this pH range, which were determined to be 4.5 ± 0.1 × 10^−5^·s^−1^ at 25 °C and 7.30 ± 0.04 × 10^−4^ s^−1^ at 50 °C. The corresponding half-life times are 258 min at 25 °C and 19 min at 50 °C. Thus, the [CuL^2^]^2+^ complex experiences significant hydrolysis in aqueous media at room temperature. Analogous studies performed for the [CuL^4^]^2+^ complex provided rate constants of 3.12 ± 0.01 × 10^−9^·s^−1^ and 1.265 ± 0.002 × 10^−7^·s^−1^ at 25 and 50 °C, respectively, which correspond to half-life times of ~89 (25 °C) and ~2 (50 °C) months. Thus, the replacement of the 1-(3-nitrophenyl)-3-phenylurea unit of L^2^ by a 1-ethyl-3-(3-nitrophenyl)urea group increases the stability toward hydrolysis of the corresponding Cu^2+^ complex by 3–4 orders of magnitude.

Most of the studies regarding the decomposition of ureas focused on its hydrolysis to ammonium and carbamate ions [66,67] (the latter is unstable and its decomposition gives rise to the formation of ammonium ions, carbon dioxide, and water [68]) by urease [69]. Kinetic experiments performed in water at pH 7 and 38 °C showed that the value of *k*_obs_ for this reaction decreases as the substitution on the urea moiety increases (from 5913 s^−1^ for urea to 0.075 s^−1^ for *N*-methylurea [70,71]). The value reported for the uncatalyzed hydrolysis of tetramethylurea (6.0×10^−11^ s^−1^) points to a very slow reaction in the absence of urease [72]. Thus, we conclude that the relatively high rate constants determined for the hydrolysis of the urea groups in [ZnL^1^]^2+^, [ZnL^2^]^2+^, and [CuL^2^]^2+^ are related to the coordination of a nitrogen atom of the urea unit to the metal ion.

### 2.4. Binding to Phosphorylated Anions

**Figure 4:**
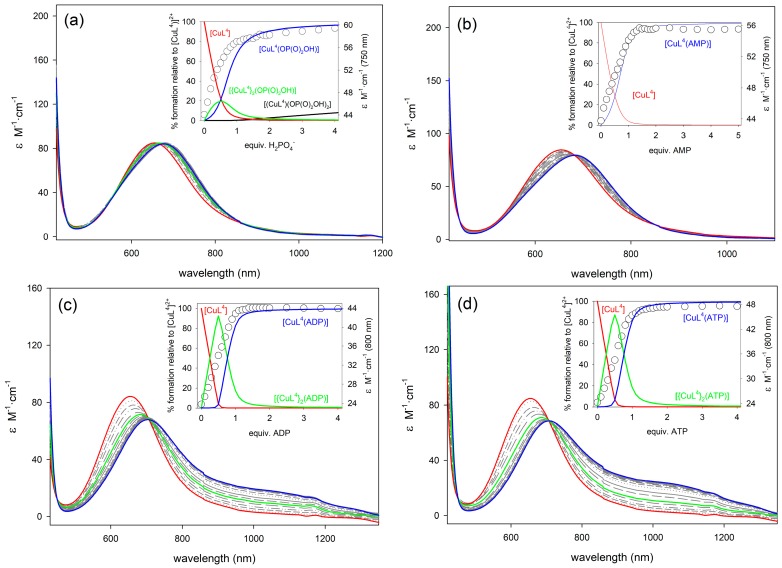
Family of UV/vis spectra taken during the course of the titration of [CuL^4^]^2+^ (5 × 10^−3^ M in H_2_O, pH 7.0, MOPS 0.1 M, 25 °C) with standard solutions (0.5 M) of: (**a**) NaH_2_PO_4_; (**b**) Na_2_AMP; (**c**) Na_2_ADP; and, (**d**) Na_2_ATP. Insets: titration profiles at selected wavelengths *vs* equivalents of anion and species distribution diagram.

The binding of phosphorylated anions phosphate, AMP, CMP, UMP, PPi, ADP, and ATP to [CuL^3^](ClO_4_)_2_, [CuL^4^](ClO_4_)_2_, and [CuL^5^](ClO_4_)_2_ was investigated by using spectrophotometric titrations in aqueous solutions (pH = 7.0, MOPS buffer). The absorption spectra of the complexes show a *d*-*d* absorption band with a maximum at 615 ([CuL^3^]^2+^), 654 ([CuL^4^]^2+^), and 643 nm ([CuL^5^]^2+^), typical of *d*_xz_, *d*_yz_ → *d*_x^2^−y^2^_ transitions in a tetragonal ligand field [73]. This is consistent with the Jahn-Teller distorted octahedral environment, where two coordination positions are likely occupied by water molecules, and supported by the molar conductivity values of these complexes in methanol solution. Addition of phosphate, AMP, CMP, or UMP causes slight changes in the *d*-*d* absorption band of the complexes, in particular, a slight bathochromic shift of the absorption maximum and small changes in the molar extinction coefficients (Figure 4, see also Figures S6–S8, Supplementary Materials). This indicates that the complexes retain the distorted octahedral coordination upon anion binding. A completely different situation is however observed upon addition of PPi, ADP, and ATP, as these anions provoke both an important bathochromic shift of the *d*-*d* absorption band and the appearance of a shoulder in the low energy side. This is characteristic of Cu^2+^ complexes having trigonal bipyramidal coordination geometries, which in the case of [CuL^2^]^2+^ complexes, was related to the de-coordination of the weakly bonded urea nitrogen atom [49]. This is in line with the X-ray structure of [{ZnL^4^}_2_(μ-PPi)] shown in Figure 2, in which the anion coordinates to the metal ion in a bidentate fashion and establishes hydrogen-bonding interactions with the urea group.

The titration profiles obtained with AMP, CMP, and UMP were satisfactory analyzed using a 1:1 binding model, providing the association constants listed in Table 3. In the case of PPi, ADP and ATP satisfactory fittings required an equilibrium model, including both 2:1 and 1:1 species ([CuL^n^]^2+^: Anion, *n* = 3, 4 or 5). The high resolution mass spectra recorded by electrospray ionization (ESI^+^) for solutions of [CuL^4^]^2+^ in the presence of one equivalent of PPi, ADP, or ATP show peaks due to the 2:1 and 1:1 entities (Table S1 and Figure S9, Supplementary Materials), which supports the model used for the determination of association constants. A similar situation was observed for phosphate, which also forms 2:1 and 1:1 complexes. The association constants obtained from the analysis of the titration curves are reported in Table 3. The anions containing a single phosphate unit (phosphate, AMP, CMP, and UMP) provide similar Log *K*_11_ values, which show that the nature of the nucleobase does not affect significantly the association constants. Thus, most likely the nucleobase is not participating significantly in the recognition process as observed for CMP and UMP in previous studies [28]. In the case of AMP, similar values of the stability constants were obtained for the corresponding adducts, so the formation of a macrochelate ring involving both the phosphate and the nitrogen N-7 of the adenosine moiety does not seem to take place. This could be related to the arrangement of the ligands around the Cu^2+^ ion in the complexes, which may block the effective approach of the nitrogen N-7 of adenosine to the inner sphere of the Cu^2+^ ion, thus inhibiting its coordination [29].

**Figure 5:**
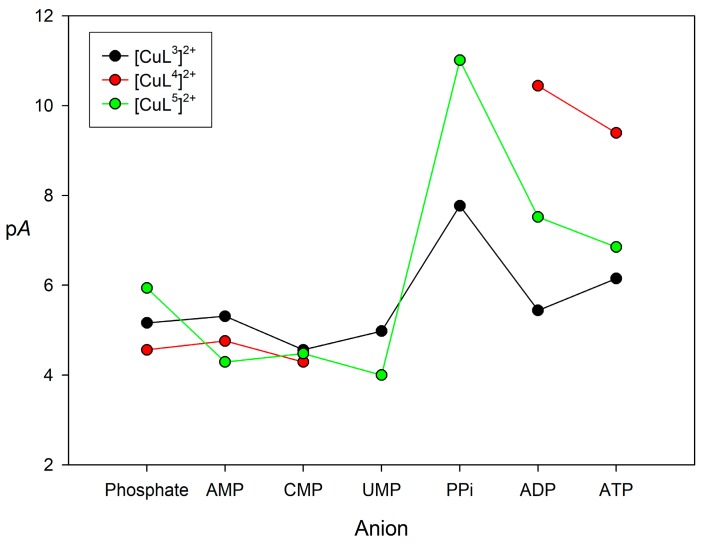
p*A* values (p*A*= −log[Anion]_free_, with [Anion] = 0.001 M and [(CuL^n^)^2+^] = 0.01 M and pH 7.0) calculated for the association of [CuL^n^]^2+^ complexes (*n* = 3, 4 or 5) with phosphorylated anions with the equilibrium constants listed in Table 3.

The comparison of association constants obtained with different equilibrium models may be misleading. Thus, we used p*A* values defined as p*A* = −log[Anion]_free_ using an anion concentration of 0.001 M and a concentration of [CuL^n^]^2+^ complex of 0.01 M (pH 7.0). The definition of p*A* is similar to that of p*M* often used to assess the relative stabilities of metal complexes in aqueous media [74]. The p*A* values obtained for all of the monophosphate anions are rather similar (Figure 5, see also Table 3), with no particular selectivity for any of them being observed. When considering the spectral changes that were displayed during the titrations, this suggests monodentate coordination of the phosphate anion to the metal center in the 1:1 complexes. The values obtained for the anions containing two or three phosphate units are clearly higher, particularly in the case of [CuL^4^]^2+^ and [CuL^5^]^2+^. This can be attributed, at least in part, to a bidentate coordination of the anion to the metal center, which for [CuL^3^]^2+^ and [CuL^4^]^2+^ triggers the de-coordination of the urea nitrogen atom. However, the nature of the receptor results in rather different stabilities. The p*A* values obtained for [CuL^3^]^2+^ and [CuL^5^]^2+^ show a similar trend, with the highest stability being observed for PPi. However, [CuL^5^]^2+^ shows higher p*A* values than [CuL^3^]^2+^, perhaps because of the more open structure of [CuL^5^]^2+^ related to the absence of the urea group. The high selectivity of [CuL^5^]^2+^ for PPi over ADP and ATP suggests that the *cis*-diol groups of the nucleotides are not forming esters with the phenylboronic acid moiety of the ligand.

Unfortunately, the addition of PPi to aqueous solutions of [CuL^4^]^2+^ caused the precipitation of the 2:1 aduct, which is subsequently redissolved upon addition of an excess of the anion. This prevented us from determining the corresponding association constants. Nevertheless, the p*A* values characterizing the association of [CuL^4^]^2+^ with ADP and ATP are clearly higher than those that were obtained for [CuL^5^]^2+^ and [CuL^3^]^2+^. We attribute this to the electron withdrawing effect of the –NO_2_ group of L^4^, which results in: (1) A weaker coordination of the urea group to the metal ions, so that it can be more easily displaced by anion coordination; and (2) A cooperative binding through coordination of the anion to the metal center and hydrogen-bonding interactions involving the urea NH donors and oxygen atoms of the anion (as evidenced in the X-ray structures shown in Figure 2). These interactions are likely weaker in the case of [CuL^3^]^2+^, and might be additionally hindered by the presence of the boronic group in position 2 with respect to the urea unit.

In the case of [CuL^3^]^2+^ and [ZnL^3^]^2+^ complexes, the high resolution mass spectra recorded in dmso in the presence of one equivalent of AMP, ADP, and ATP show peaks due to 1:1 entities (Figure S10, Supplementary Materials). The position and isotopic profiles of the peaks indicate that the phenylboronic moiety has reacted with the *cis*-diol units present in the sugar ring of nucleotides in all cases. Similar measurements performed in aqueous solution did not evidence the formation of phenylboronic esters in the recognition of the nucleotides in this medium, suggesting that this cooperative interaction is not present in aqueous solution.

### 2.5. NMR Studies

**Figure 6:**
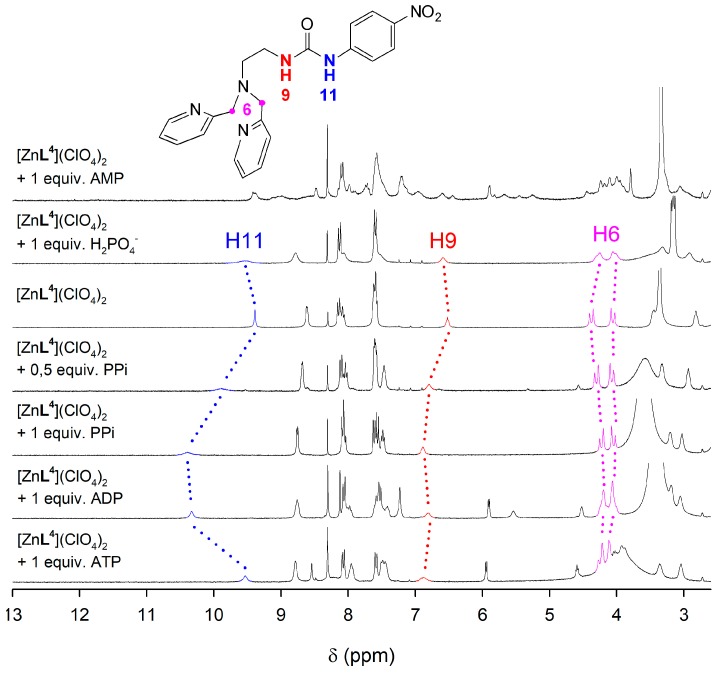
^1^H-NMR spectra (300 MHz, 298 K) recorded for the [ZnL^4^]^2+^ complex in the presence of different anions as their sodium salts in dmso-*d*_6_.

The interaction of the [ZnL^4^]^2+^ complex with phosphorylated anions was investigated by using ^1^H-NMR spectroscopy in dmso-*d*_6_ solution, which was selected as solvent in view of the low solubility of the complex in aqueous media. The ^1^H-NMR signals of the urea protons were particularly informative with respect to the interaction of the anion with the complex (Figure 6). These signals are observed at 9.40 and 6.52 ppm in the spectrum of the [ZnL^4^]^2+^ complex, dissolved as the perchlorate salt. The addition of phosphate or AMP does not provoke significant changes in the chemical shifts of the urea protons. However, the signal at 9.40 ppm experiences important shifts to lower fields upon addition of one equivalent of PPi or ADP, shifting to 10.40 and 10.33 ppm, respectively. In parallel, the signal at 6.52 ppm undergoes smaller downfield shifts to 6.89 and 6.81 ppm, respectively. These spectral changes reflect the establishment of hydrogen-bonding interactions involving the urea fragment and the anion [75]. Interestingly, the addition of ATP provokes smaller shifts of the signals of urea protons to 9.53 and 6.88 ppm, which is in line with the lower p*A* value determined for the association of ATP and [CuL^4^]^2+^. A similar behavior is also observed for the [ZnL^3^]^2+^ complex, which contains a phenylboronic function (Figure S11, Supplementary Materials).

**Figure 7:**
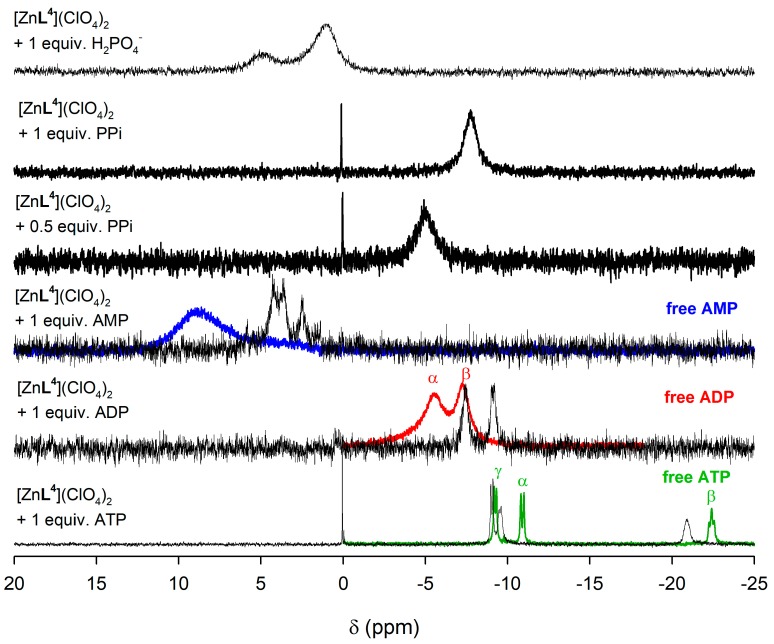
^31^P NMR spectra (500 MHz, 298 K) recorded for [ZnL^4^]^2+^ complex in the presence of AMP, ADP, ATP and phosphate as their sodium salts in dmso-*d*_6_. The ^31^P NMR spectra of free metabolites are shown as colored traces.

The corresponding ^31^P NMR spectrum of the ATP adduct (Figure 7) shows three signals at −9.55, −20.92 and −9.05 ppm due to the P_α_, P_β_, and P_γ_ nuclei, respectively. The corresponding chemical shifts measured for the uncoordinated ATP anion are −10.90, −22.42, and −9.26 ppm. The small chemical shift induced by coordination in the signal of P_γ_ and the relatively large chemical shifts experienced by P_α_ and P_β_ indicates that only the two inner phosphate groups of ATP play a significant role in the coordination to the metal ion. In the case of ADP coordination to the metal ion induces important upfield shifts of the signals of both P_α_ and P_β_, from −7.29 to −9.13 ppm (P_α_) and from −5.56 to −7.46 ppm (P_β_). Taken together, these results indicate that both ADP and ATP coordinate to the metal ion through the phosphate units containing P_α_ and P_β_, likely in a similar way as observed in the solid state for [{ZnL^4^}_2_(μ-PPi)] and [{CuL^4^}_2_(μ-PPi)] (Figure 2). It is also worth noting that the spectrum recorded in the presence of ATP of PPi shows a small signal at 0 ppm due to the presence of phosphoric acid, indicating that the complex catalyzes the hydrolysis of the PPi anion and the triphosphate unit of ATP. In the case of [ZnL^3^]^2+^, similar ^31^P chemical shifts are observed, but the higher relative intensity of the signal at 0 ppm for polyphosphorylated anions indicates a higher extent of the hydrolysis process (Figure S12, Supplementary Materials).

The ^31^P NMR spectra recorded in the presence of phosphate shows two very broad signals, which evidences the presence of different species in solution involved in dynamic processes. In the case of AMP, the ^31^P NMR spectra show at least three signals for both [ZnL^3^]^2+^ and [ZnL^4^]^2+^ complexes, none of which coincides with that of free AMP, again suggesting the presence of several species in solution.

To check whether the structures in solution of [ZnL^4^(H_2_PPi)] and [{ZnL^4^}_2_(μ-PPi)] complexes correspond to those observed in solid state, the geometries of both monomeric and dimeric species were modeled by means of DFT calculations (TPSSh/SVP) in dmso solution (Figure S13, Supplementary Materials). In both cases, the possibility that the pyrophosphate anion was totally deprotonated or doubly protonated was considered, and the corresponding ^31^P NMR spectra of the four minimum energy geometries were calculated at the TPSSh/TZVP level (Table 4). The minimum energy geometry obtained for the [{ZnL^4^}_2_(μ-PPi)] species is in agreement with the one previously described in the solid state, where identical conformations of the ligands backbone (‘bent propeller’), coordination polyhedron around the Zn(II) metal ion (trigonal bipyramid), and intramolecular hydrogen-bonding interactions are present. In the case of the [ZnL^4^(H_2_PPi)] species, the obtained geometry differs considerably from that observed for the [CuL^4^(H_2_PPi)] complex in the solid state. The coordination polyhedron around the Zn^2+^ metal ion can be now best described as a square pyramid, with the ligand in an ‘open wing butterfly‘ conformation. Despite this, it is noticeable to remark that in both geometries the pyrophosphate anion is coordinated to the Zn(II) metal ions forming six-membered chelate rings in κ^2^-H_2_PPi^2−^ or μ_2_κ^4^-PPi^4−^ modes for [ZnL^4^(μ-PPi)] and [{ZnL^4^}_2_(μ-PPi)], respectively. Hydrogen bond interactions between the oxoanion and the urea moieties are observed in both cases.

The comparison of the calculated chemical shifts with the experimental values recorded in the presence of 0.5 or 1 equivalents of pyrophosphate anion are in agreement with those calculated for a fully deprotonated form in the case of the [{ZnL^4^}_2_(μ-PPi)] dimeric complex, and with a doubly protonated form for the [ZnL^4^(H_2_PPi)] monomeric complex. The good agreement between experimental and calculated data suggests that both complexes adopt the calculated minimum energy geometries in solution.

## 3. Materials and Methods

### 3.1. General Methods

Elemental analyses were carried out on a ThermoQuest Flash EA 1112 elemental analyzer. ESI-TOF low resolution mass spectra (MS) were recorded from MeOH/MeCN/CHCl_3_, MeOH/MeCN, MeCN/CHCl_3_, or MeCN solutions, while the high resolution MS were recorded from MeCN/H_2_O or MeCN/dmso solutions, in both cases using a LC-Q-q-TOF Applied Biosystems QSTAR Elite spectrometer in the positive mode. IR spectra were recorded using a Bruker Vector 22 spectrophotometer equipped with a Golden Gate attenuated total reflectance (ATR) accessory (Specac). ^1^H, ^13^C and ^31^P NMR spectra were recorded at 25 °C on Bruker Avance 300 and Bruker Avance 500 spectrometers, and spectral assignments were based in part on 2D COSY, HSQC, and HMBC experiments. UV/vis spectra were recorded with a Perkin-Elmer Lambda 900 spectrophotometer. The spectra obtained from solutions were recorded with quartz cells (path length: 1 cm) and the cell holder was thermostatted at 25.0 °C, through circulating water. Anion binding studies were performed by monitoring the spectral changes of 10^−3^ M solutions of [CuL^3^](ClO_4_)_2_ or [CuL^5^](ClO_4_)_2_ complexes in H_2_O at pH 7.0 upon addition of a 0.1 M solution of the corresponding sodium salt containing a 10^−3^ M concentration of the corresponding complex at pH 7.0. For the [CuL^4^](ClO_4_)_2_ complex, the concentrations of the titration solutions and the solutions of the sodium salts were 5 × 10^−3^ M and 0.5 M, respectively. The pH was buffered with MOPS in all cases. Binding constants were obtained by using a simultaneous fit of the UV/vis absorption spectral changes at 7–12 selected wavelengths in the range 500–1200 nm. A minimum of 26 absorbance data points at each of these wavelengths was used, and all spectrophotometric titration curves were fitted with the HYPERQUAD 2008 (HypSpec) program [76].

#### Reagents

(3-Aminophenyl)boronic acid (**1**), 3-tolylboronic acid (**2**), 2-bromoethanamine hydrobromide (**11**), ethane-1,2-diol, di-*tert*-butyl dicarbonate, bis(pyridin-2-ylmethyl)amine, 1-isocyanato-4-nitrobenzene, and 1-chloro-2-isocyanatoethane were obtained from commercial sources and used as received. Solvents were of reagent grade and used without further purification.

***Caution:*** Although we have experienced no difficulties with the perchlorate salts, these should be regarded as potentially explosive and handled with care [77].

### 3.2. Synthesis

*3-(1,3,2-Dioxaborolan-2-yl)aniline* (**3**). A solution of (3-aminophenyl)boronic acid (**1**) (2.536 g, 16.04 mmol) and ethane-1,2-diol (1.169 mL, 20.85 mmol) in acetonitrile (50 mL) was heated to reflux with stirring for 6 h. The solvent was removed by rotary evaporation and the resulting oil was dried under vacuum, after which it became a pale brown solid (**3**) that was used without further purification (2.605 g, 100%). *δ*_H_ (solvent CDCl_3_, 500 MHz, 298 K): 7.23–7.17 (m, 2H), 7.14 (m, 1H), 6.80 (ddd, 1H, ^3^*J* = 7.7 Hz, ^4^*J* = 2.6 Hz, ^4^*J* = 1.4 Hz), 4.35 (s, 4H), 3.49 (s, 2H). *δ*_C_ (solvent CDCl_3_, 125.8 MHz, 298 K): 145.8, 128.9, 125.2, 121.3, 118.4, 66.1. MS-ESI^+^, *m*/*z* (%BPI): [**3** + H]^+^, 164.1 (47%). Elem. Anal. Calcd for C_8_H_10_BNO_2_: C, 59.0; H, 6.2; N, 8.6%. Found: C, 58.6; H, 6.3; N, 8.5%. IR: 3444, 3361, 3215 ν(N-H), 2981, 2910 ν(C-H), 1625, 1579, 1484, 1443 ν(C=C), 1338 ν(C-BO_2_), 1210 ν(O-B-O) cm^−1^.

*2-(3-Tolyl)-1,3,2-dioxaborolane* (**4**). This compound was prepared by adapting the experimental procedure found in the literature [53]. A solution of 3-tolylboronic acid (**2**) (1.829 g, 13.05 mmol) and ethane-1,2-diol (0.951 mL, 16.96 mmol) in toluene (50 mL) was heated to reflux with stirring for 16 h using a Dean-Stark apparatus for water removal. The resulting solution was filtered while hot and the filtrate concentrated to dryness. The residue was purified by column chromatography on SiO_2_ with CH_2_Cl_2_ as the eluent to give pure product **4** as a pale yellow oil (1.691 g, 80%). *δ*_H_ (solvent CDCl_3_, 500 MHz, 298 K): 7.67 (s, 1H), 7.65 (m, 1H), 7.32–7.31 (m, 2H), 4.37 (s, 4H), 2.39 (s, 3H). *δ*_C_ (solvent CDCl_3_, 125.8 MHz, 298 K): 137.3, 135.6, 132.4, 131.9, 127.9, 66.1, 21.4. MS-ESI^+^, *m*/*z* (%BPI): [**4** + H]^+^, 163.1 (38%). Elem. Anal. Calcd for C_9_H_11_BO_2_: C, 66.7; H, 6.8%. Found: C, 66.6; H, 6.8%. IR: 3023, 2974, 2908 ν(C-H), 1607, 1583, 1481 ν(C=C), 1335 ν(C-BO_2_), 1206 ν(O-B-O) cm^−1^.

*1-(3-(1,3,2-Dioxaborolan-2-yl)phenyl)-3-(2-(chloromethyl)phenyl)urea* (**5**). A solution of compound **3** (1.400 g, 8.590 mmol) and 1-(chloromethyl)-2-isocyanatobenzene (1.184 mL, 8.590 mmol) in diethyl ether (40 mL) was stirred at room temperature for 16 h. The precipitate formed was isolated by filtration and washed with diethyl ether (3 × 5 mL) to give 2.594 g of the desired compound (91%) as a white solid. *δ*_H_ (solvent dmso-*d*_6_, 500 MHz, 298 K): 9.38 (s), 9.20 (s), 8.25 (s), 8.21 (s), 7.88 (m), 7.69–7.64 (m), 7.61 (m), 7.43 (m), 7.34–7.31 (m), 7.27–7.19 (m), 7.06 (m), 4.85 (s), 4.35 (s), 4.32 (s), 3.39 (s). *δ*_C_ (solvent dmso-*d*_6_, 125.8 MHz, 298 K): 152.7, 139.4, 137.5, 130.6, 129.2, 128.5, 127.9, 127.6, 124.0, 123.1, 122.4, 121.1, 65.9, 65.7, 62.8, 43.5. MS-ESI^+^, *m*/*z* (%BPI): [**5** + H]^+^, 331.1 (7%); [**5** − PG + H]^+^, 305.1 (23%); [**5** − Cl]^+^, 295.1 (9%); [**5** − PG − Cl]^+^, 269.1 (85%). Elem. Anal. Calcd for C_16_H_16_BClN_2_O_3_: C, 58.1; H, 4.9; N, 8.5%. Found: C, 58.4; H, 4.9; N, 8.3%. IR: 3285 ν(N-H), 2979, 2912 ν(C-H), 1637 ν(C=O), 1606, 1586, 1489, 1477 ν(C=C), 1560 δ(N-H), 1335 ν(C-BO_2_), 1214 ν(O-B-O), 672 ν(C-Cl) cm^−1^.

*1-(3-(1,3,2-Dioxaborolan-2-yl)phenyl)-3-(2-chloroethyl)urea* (**7**). A solution of compound **3** (1.400 g, 8.590 mmol) and 1-chloro-2-isocyanatoethane (0.755 mL, 8.590 mmol) in diethyl ether (40 mL) was stirred at room temperature for 16 h. The precipitate formed was isolated by filtration and washed with diethyl ether (3 × 5 mL) to give 2.052 g of the desired compound (89%) as a white solid. *δ*_H_ (solvent dmso-*d*_6_, 500 MHz, 298 K): 8.70 (s, 1H), 7.76 (m, 1H), 7.55−7.52 (m, 1H), 7.27–7.25 (m, 2H), 6.39 (t, 1H, ^3^*J* = 5.7 Hz), 4.31 (s, 4H), 3.66 (t, 2H, ^3^*J* = 6.2 Hz), 3.42 (c, 2H, ^3^*J* = 6.0 Hz). *δ*_C_ (solvent dmso-*d*_6_, 125.8 MHz, 298 K): 155.1, 155.0, 139.9, 139.4, 128.4, 127.7, 127.4, 127.2, 123.8, 123.7, 120.9, 119.8, 65.6, 62.8, 44.5, 44.4, 41.2. MS-ESI^+^, *m*/*z* (%BPI): [**7** + H]^+^, 269.1 (48%); [**7** − PG + H]^+^, 243.1 (100%). Elem. Anal. Calcd for C_11_H_14_BClN_2_O_3_: C, 49.2; H, 5.3; N, 10.4%. Found: C, 49.3; H, 5.2; N, 10.4%. IR: 3329 ν(N-H), 2978, 2914 ν(C-H), 1629 ν(C=O), 1605, 1486, 1476 ν(C=C), 1564 δ(N-H), 1333 ν(C-BO_2_), 1214 ν(O-B-O), 614 ν(C-Cl) cm^-1^.

*2-(3-(Bromomethyl)phenyl)-1,3,2-dioxaborolane* (**9**). This compound was prepared by adapting the experimental procedure found in the literature [53]. A mixture of compound **4** (1.570 g, 9.693 mmol), NBS (1.830 g, 10.18 mmol) and PDB (0.056 g, 0.173 mmol) in CCl_4_ (40 mL) was heated to reflux with stirring for 16 h. The reaction mixture was allowed to cool to room temperature, the precipitated succinimide was removed by filtration and the filtrate was concentrated under reduced pressure. The residue was purified by column chromatography on SiO_2_ with CH_2_Cl_2_ as the eluent to give pure product **9** as a pale yellow oil (1.039 g, 45%). *δ*_H_ (solvent CDCl_3_, 500 MHz, 298 K): 7.84 (m, 1H), 7.74 (m, 1H), 7.51 (m, 1H), 7.37 (t, 1H, ^3^*J* = 7.5 Hz), 4.51 (s, 2H), 4.39 (m, 4H). *δ*_C_ (solvent CDCl_3_, 125.8 MHz, 298 K): 136.3, 135.5, 134.9, 132.3, 128.5, 66.2, 33.6. MS-ESI^+^, *m*/*z* (%BPI): [**9** − Br]^+^, 161.1 (100%). Elem. Anal. Calcd for C_9_H_10_BBrO_2_: C, 44.9; H, 4.2%. Found: C, 45.0; H, 4.3%. IR: 2976, 2908 ν(C-H), 1605, 1583 ν(C=C), 1338 ν(C-BO_2_), 1216 ν(O-B-O), 602 ν(C-Br) cm^−1^.

*tert-Butyl-(2-bromoethyl)carbamate* (**12**). This compound was prepared according to the literature procedure [78]. A suspension of compound **11** (2.500 g, 12.08 mmol) in 40 mL of dioxane was stirred at 0 °C for 30 min, and subsequently a solution of di-*tert*-butyl dicarbonate (2.663 g, 12.08 mmol) and triethylamine (1.701 mL, 12.08 mmol) in 15 mL of dioxane was added slowly. The mixture was stirred at 0 °C for two hours, and at room temperature for two days. The solid formed was filtered and the filtrate concentrated to dryness. The resulting oil was dissolved in chloroform (50 mL) and washed with water (3 × 15 mL). The organic layer was dried over anhydrous sodium sulfate and the solvent removed by rotary evaporation. The residue was dried under vacuum for several hours to give 2.152 g (80%) of **12** as a pale yellow oil. *δ*_H_ (solvent CDCl_3_, 500 MHz, 298 K): 5.02 (s, 1H), 3.50 (m, 2H), 3.42 (m, 2H), 1.42 (s, 9H). *δ*_C_ (solvent CDCl_3_, 125.8 MHz, 298 K): 155.7, 79.9, 42.5, 32.8, 28.4. MS-ESI^+^, *m*/*z* (%BPI): [**12** + Na]^+^, 246.0 (1%); [**12** − Boc + H]^+^, 124.0 (68%). Elem. Anal. Calcd for C_7_H_14_BrNO_2_: C, 37.5; H, 6.3; N, 6.3%. Found: C, 37.1; H, 5.7; N, 6.8%. IR: 3336 ν(N-H), 2977, 2932 ν(C-H), 1689 ν(C=O), 1507 ν(N-H), 1162 ν_a_(N-C) cm^−1^.

*tert-Butyl-(2-(bis(pyridin-2-ylmethyl)amino)ethyl)carbamate* (**13**). A solution of compound **12** (2.030 g, 9.060 mmol), bis(pyridin-2-ylmethyl)amine (1.528 mL, 8.237 mmol), *N*,*N*-diisopropylethylamine (2.898 mL, 16.47 mmol) and a catalytic amount of KI in acetonitrile (100 mL) was heated to reflux with stirring for three days. The resulting solution was filtered while hot and the filtrate concentrated to dryness. The residue was extracted with chloroform (5 × 50 mL) and water (25 mL), the organic layers combined and dried over anhydrous sodium sulfate, and the solvent evaporated under reduced pressure. The resulting oil was purified on a *CombiFlash RF-200*, using a 48 g-neutral alumina column and CH_2_Cl_2_ as eluent to obtain pure product **13** as an orange oil which was dried under vacuum for several hours (1.092 g, 39%). *δ*_H_ (solvent CDCl_3_, 500 MHz, 298 K): 8.51 (m, 2H), 7.60 (m, 2H), 7.38 (d, 2H, ^3^*J* = 7.8 Hz), 7.12 (m, 2H), 5.80 (s, 1H), 3.83 (s, 4H), 3.20 (m, 2H), 2.67 (m, 2H), 1.41 (s, 9H). *δ*_C_ (solvent CDCl_3_, 125.8 MHz, 298 K): 159.3, 156.3, 149.2, 136.6, 123.2, 122.2, 78.8, 60.3, 53.7, 38.6, 28.6. MS-ESI^+^, *m*/*z* (%BPI): [**13** + Na]^+^, 365.2 (100%); [**13** + H]^+^, 343.2 (19%). Elem. Anal. Calcd for C_19_H_26_N_4_O_2_: C, 66.6; H, 7.7; N, 16.4%. Found: C, 66.3; H, 7.7; N: 15.6%. IR: 3315 ν(N-H), 3051–2821 ν(C-H), 1699 ν(C=O), 1590, 1569 ν(Py), 1520 ν(N-H), 1167 ν_a_(N-C), 757 ν(C-H) cm^−1^.

*N′,N′-Bis(pyridin-2-ylmethyl)ethane-1,2-diamine* (**14**). A solution of compound **13** (1.075 g, 3.139 mmol) and trifluoroacetic acid (10 mL, 129.3 mmol) in chloroform (10 mL) was stirred at room temperature for 16 h. The solvent was evaporated under reduced pressure and the residue was dissolved in chloroform (30 mL). The acid excess was neutralized with an aqueous solution of NaOH (1 M) and the organic layer washed with distilled water (2 × 15 mL). The organic phase was dried over anhydrous sodium sulfate and the solvent removed by rotary evaporation to give the pure product **14** as an orange oil that was dried under vacuum for several hours (0.727 g, 96%). *δ*_H_ (solvent CDCl_3_, 500 MHz, 298 K): 8.50 (m, 2H), 7.62 (m, 2H), 7.45 (d, 2H, ^3^*J* = 7.8 Hz), 7.12 (m, 2H), 3.82 (s, 4H), 2.77 (t, 2H, ^3^*J* = 6.1 Hz), 2.65 (t, 2H, ^3^*J* = 6.0 Hz), 2.06 (s, 2H). *δ*_C_ (solvent CDCl_3_, 125.8 MHz, 298 K): 159.7, 149.2, 136.5, 123.1, 122.1, 60.8, 57.4, 39.7. MS-ESI^+^, *m*/*z* (%BPI): [**14** + H]^+^, 243.2 (100%). Elem. Anal. Calcd for C_14_H_18_N_4_: C, 69.4; H, 7.5; N, 23.1%. Found: C, 69.1; H, 6.9; N, 22.8%. IR: 3360 ν(N-H), 3054–2823 ν(C-H), 1589, 1568, 1473, 1432 ν(Py), 756 ν(C-H) cm^−1^.

*(3-(3-(2-((Bis(pyridin-2-ylmethyl)amino)methyl)phenyl)ureido)phenyl)boronic acid* (**L^1^**). A solution of compound **5** (1.150 g, 3.479 mmol), bis(pyridin-2-ylmethyl)amine (0.587 mL, 3.163 mmol), *N*,*N*-diisopropylethylamine (1.113 mL, 6.325 mmol) and a catalytic amount of KI in acetonitrile (50 mL) was heated to reflux with stirring for three days. The resulting solution was filtered while hot and the filtrate concentrated to dryness. The residue was extracted with chloroform (5 × 50 mL) and water (25 mL), the organic extracts combined and dried over anhydrous sodium sulfate, and the solvent evaporated under reduced pressure. The resulting oil was purified on a *CombiFlash RF-200*, using a 48 g-neutral alumina column and CHCl_3_/MeOH as eluent (from 0 to 3%; the desired product eluted from 2 to 3%) to obtain pure product **L^1^** as a pale brown solid (0.607 g, 40%). *δ*_H_ (solvent dmso-*d*_6_, 500 MHz, 298 K): 10.30 (s, 1H, H14), 8.96 (s, 1H, H16), 8.50 (*ddd*, 2H, H1, ^3^*J* = 4.8 Hz, ^4^*J* = 1.7 Hz, ^4^*J* = 0.8 Hz), 8.03 (m, H12), 8.01 (s, H22), 7.77 (m, 1H, H23), 7.76–7.73 (m, H20), 7.71 (td, H3, ^3^*J* = 7.7 Hz, ^4^*J* = 1.8 Hz), 7.46 (m, 1H, H18), 7.40 (m, 2H, H4), 7.30 (t, 1H, H19, ^3^*J* = 7.7 Hz), 7.27–7.20 (m, 5H, H2, H9 and H11), 6.93 (td, 1H, H10, ^3^*J* = 7.4 Hz, ^4^*J* = 1.2 Hz), 3.80 (s, 4H, H6), 3.72 (s, 2H, H7). (See Figure S14 in Supplementary Materials for labelling scheme of the ligand). *δ*_C_ (solvent dmso-*d*_6_, 125.8 MHz, 298 K): 157.7 C5, 152.6 C15, 149.0 C1, 139.4 C13, 139.2 C17, 136.9 C3, 129.9 C9, 127.9 C19, 127.9 C18, 127.8 C11, 125.6 C8, 124.2 C23, 123.6 C4, 122.5 C2, 121.5 C10, 120.2 C20, 119.4 C12, 58.5 C6, 56.4 C7. MS-ESI^+^, *m*/*z* (%BPI): [**L^1^** + H]^+^, 468.2 (100%). Elem. Anal. Calcd for C_26_H_26_BN_5_O_3_: C, 64.3; H, 5.8; N, 14.4%. Found: C, 64.5; H, 5.1; N: 14.6%. IR: 3290 ν(N-H/O-H), 3050–2830 ν(C-H), 1695 ν(C=O), 1593, 1571, 1477 ν(Ph/Py), 1538 ν(N-H), 1207 ν(O-B-O), 749 ν(C-H) cm^−1^.

*(3-(3-(2-(Bis(pyridin-2-ylmethyl)amino)ethyl)ureido)phenyl)boronic acid* (**L^3^**). A solution of compound **7** (0.350 g, 1.303 mmol), bis(pyridin-2-ylmethyl)amine (0.220 mL, 1.185 mmol) and *N*,*N*-diisopropylethylamine (0.417 mL, 2.370 mmol) in acetonitrile (30 mL) was heated to reflux with stirring for four days. The resulting solution was filtered while hot and the filtrate concentrated to dryness. The residue was extracted with chloroform (5 × 20 mL) and water (10 mL). During this process a brown solid insoluble in both phases was formed. This solid was filtered off, the organic extracts were combined, dried over anhydrous sodium sulfate and the solvent evaporated under reduced pressure. The resulting oil was purified on a *CombiFlash RF-200*, using an 8 g-neutral alumina column and CH_2_Cl_2_/MeOH as eluent (from 0 to 5%; the desired product eluted from 3 to 5%) to obtain pure product **L^3^** as a pale yellow solid (0.113 g, 24%). *δ*_H_ (solvent dmso-*d*_6_, 500 MHz, 298 K): 8.55 (s, 1H, H11), 8.48 (m, 2H, H1), 7.96 (s, 2H, H17), 7.72 (td, 2H, H3, ^3^*J* = 7.7 Hz, ^4^*J* = 1.7 Hz), 7.60–7.56 (m, 4H, H4, H15 and H18), 7.33 (m, 1H, H13), 7.23 (m, 2H, H2), 7.18 (t, 1H, H14, ^3^*J* = 7.6 Hz), 6.15 (m, 1H, H9), 3.81 (s, 4H, H6), 3.25 (c, 2H, H8, ^3^*J* = 5.9 Hz), 2.59 (t, 2H, H7, ^3^*J* = 6.0 Hz). (See Figure S14 in Supplementary Materials for labelling scheme of the ligand). *δ*_C_ (solvent dmso-*d*_6_, 125.8 MHz, 298 K): 159.0 C5, 155.3 C10, 148.8 C1, 139.7 C12, 136.5 C3, 134.7 C16, 127.7 C14, 127.0 C13, 123.8 C15, 122.8 C4, 122.2 C2, 119.8 C18, 59.6 C6, 53.8 C7, 37.0 C8. MS-ESI^+^, *m*/*z* (%BPI): [**L^3^** + H]^+^, 406.2 (100%). Elem. Anal. Calcd for C_21_H_24_BN_5_O_3_: C, 62.2; H, 6.0; N, 17.3%. Found: C, 61.9; H, 6.1; N: 17.7%. IR: 3303 ν(N-H/O-H), 3054–2849 ν(C-H), 1659 ν(C=O), 1593, 1477 ν(Ph/Py), 1551 ν(N-H), 1338 ν(C-BO_2_), 757 ν(C-H) cm^−1^.

*1-(2-(Bis(pyridin-2-ylmethyl)amino)ethyl)-3-(4-nitrophenyl)urea* (**L^4^**). A solution of compound **14** (0.713 g, 2.942 mmol) and 1-isocyanato-4-nitrobenzene (0.498 g, 2.942 mmol) in chloroform (40 mL) was stirred at room temperature for two hours. The solvent was evaporated under reduced pressure and the residue was purified on a *CombiFlash RF-200*, using a 12 g-silica gel column and CHCl_3_/MeOH as eluent (from 0 to 4% of methanol; the desired compound eluted at 4%) to give **L^4^** as a yellow oil that became a solid after remaining under vacuum for several days (1.014 g, 85%). *δ*_H_ (solvent CDCl_3_, 500 MHz, 298 K): 8.95 (s, 1H, H11), 8.46 (m, 2H, H1), 8.00 (m, 2H, H14), 7.56–7.51 (m, 4H, H3 and H13), 7.26 (d, H4, ^3^*J* = 7.8 Hz), 7.09 (m, 2H, H2), 6.99 (s, 1H, H9), 3.73 (s, 4H, H6), 3.26 (c, 2H, H8, ^3^*J* = 5.1 Hz), 2.61 (t, 2H, H7, ^3^*J* = 5.9 Hz). (See Figure S14 in Supplementary Materials for labelling scheme of the ligand). *δ*_C_ (solvent CDCl_3_, 125.8 MHz, 298 K): 158.1 C5, 155.3 C10, 149.1 C1, 146.7 C12, 141.3 C15, 136.9 C3, 125.1 C14, 123.9 C4, 122.5 C2, 117.2 C13, 60.0 C6, 53.0 C7, 37.3 C8. MS-ESI^+^, *m*/*z* (%BPI): [**L^4^** + H]^+^, 407.2 (100%). Elem. Anal. Calcd for C_21_H_22_N_6_O_3_: C, 62.1; H, 5.5; N, 20.7. Found: C, 62.4; H, 5.3; N, 20.0%. IR: 3317 ν(N-H), 3078–2804 ν(C-H), 1643 ν(C=O), 1614, 1591, 1475, 1433 ν(Ph/Py), 1553 ν_a_(NO_2_), 1328 ν_s_(NO_2_), 749 ν(C-H) cm^−1^.

*(3-((Bis(pyridin-2-ylmethyl)amino)methyl)phenyl)boronic acid* (**L^5^**). A solution of compound **9** (0.310 g, 1.287 mmol), bis(pyridin-2-ylmethyl)amine (0.217 mL, 1.170 mmol), *N*,*N*-diisopropylethylamine (0.412 mL, 2.340 mmol) and a catalytic amount of KI in acetonitrile (50 mL) was heated to reflux with stirring for 16 h. The resulting solution was filtered while hot and the filtrate concentrated to dryness. The residue was extracted with chloroform (5 × 25 mL) and water (15 mL), the organic layers combined and dried over anhidrous sodium sulfate, and the solvent evaporated under reduced pressure. The resulting oil was purified on a *CombiFlash RF-200*, using a 48 g-neutral alumina column and CH_2_Cl_2_/MeOH as eluent (from 0 to 5%; the desired product eluted from 4% to 5%) to obtain pure product **L^5^** as a pale yellow solid (0.165 g, 42%). *δ*_H_ (solvent dmso-*d*_6_, 500 MHz, 298 K): 8.48 (ddd, 2H, H1, ^3^*J* = 4.8 Hz, ^4^*J* = 1.8 Hz, ^4^*J* = 0.8 Hz), 8.04 (s, 2H, H13), 7.80 (m, H14), 7.77 (td, H3, ^3^*J* = 7.7 Hz, ^4^*J* = 1.8 Hz), 7.67 (m, 1H, H11), 7.59 (m, 2H, H4), 7.47 (m, 1H, H9), 7.31 (t, 1H, H10, ^3^*J* = 7.5 Hz), 7.24 (m, 2H, H2), 3.70 (s, 4H, H6), 3.62 (s, 2H, H7). (See Figure S14 in Supplementary Materials for labelling scheme of the ligand). *δ*_C_ (solvent dmso-*d*_6_, 125.8 MHz, 298 K): 159.2 C5, 148.8 C1, 137.5 C8, 136.6 C3, 134.6 C14, 134.1 C12, 132.9 C11, 130.4 C9, 127.4 C10, 122.5 C4, 122.2 C2, 59.1 C6, 57.9 C7. MS-ESI^+^, *m*/*z* (%BPI): [**L^5^** + Na]^+^, 356.2 (67%); [**L^5^** + H]^+^, 334.2 (100%). Elem. Anal. Calcd for C_19_H_20_BN_3_O_2_: C, 68.5; H, 6.1; N, 12.6%. Found: C, 68.2; H, 6.0; N: 11.0%. IR: 3049–2850 ν(C-H), 1593, 1571, 1475 ν(Ph/Py), 1362 ν(C-BO_2_), 758 ν(C-H) cm^−1^.

**General procedure for the preparation of [ML^1^](ClO_4_)_2_ (M = Cu or Zn).** A solution of hydrated Cu(ClO_4_)_2_ or Zn(ClO_4_)_2_ (0.103 mmol) in THF (2 mL) was added to a solution of **L^1^** (0.050 g, 0.103 mmol) in the same solvent (5 mL). The mixture was stirred at room temperature for an hour and the solvent was removed under reduced pressure. The solid was treated with diethyl ether (5 mL), filtrated and dried under vacuum.

**[CuL^1^](ClO_4_)_2_·H_2_O** ([CuL^1^](ClO_4_)_2_). Dark green solid. Yield: 0.077 g, 96%. MS-ESI^+^, *m*/*z* (%BPI): [CuL^1^(ClO_4_)]^+^, 629.1 (3%); [Cu(L^1^-H)]^+^, 529.1 (41%). Elem. Anal. Calcd for C_26_H_26_BCl_2_CuN_5_O_11_·H_2_O: C, 41.8; H, 3.8; N, 9.4%. Found: C, 42.1; H, 3.8; N, 9.1%. Λ_M_ (MeOH, 10^−3^ M, 25 °C): 154.9 cm^2^·Ω^−1^·mol^−1^ (2:1 electrolyte). IR: 3358 ν(N-H/O-H), 1651 ν(C=O), 1614 ν(Ph/Py), 1549 ν(N-H), 1345 ν(C-BO_2_), 1053 ν_a_(Cl-O), 619 ν_a_(O-Cl-O) cm^−1^. UV/vis diffuse reflectance spectroscopy: 633 nm.

**[ZnL^1^](ClO_4_)_2_** ([ZnL^1^](ClO_4_)_2_). Light brown solid. Yield: 0.073 g, 97%. MS-ESI^+^, *m*/*z* (%BPI): [ZnL^1^(ClO_4_)]^+^, 630.1 (4%); [Zn(L^1^-H)]^+^, 530.1 (100%); [ZnL^1^]^2+^, 265.6 (71%). Elem. Anal. Calcd for C_26_H_26_BCl_2_N_5_O_11_Zn: C, 42.7; H, 3.6; N, 9.6%. Found: C, 42.6; H, 3.7; N: 9.3%. Λ_M_ (MeOH, 10^−3^ M, 25 °C): 164.0 cm^2^·Ω^−1^·mol^−1^ (2:1 electrolyte). IR: 3354 ν(N-H/O-H), 1612 ν(Ph/Py), 1559 ν(N-H), 1343 ν(C-BO_2_), 1055 ν_a_(Cl-O), 620 ν_a_(O-Cl-O) cm^−1^. *δ*_H_ (solvent dmso-*d*_6_, 500 MHz, 298 K): 8.60 (m, 2H), 8.42 (s, 1H), 8.15 (s, 1H), 8.01 (s), 7.97 (m), 7.60–7.55 (m, 5H), 7.49–7.35 (m, 6H), 7.22 (m, 1H), 4.22 (d, 2H, H6A, ^2^*J* = 16.0 Hz), 3.82 (s, 2H), 3.67 (d, 2H, H6B, ^2^*J* = 16.0 Hz). *δ*_C_ (solvent dmso-*d*_6_, 125.8 MHz, 298 K): 154.4, 153.6, 147.6, 140.5, 138.6, 138.5, 134.8, 133.3, 129.4, 128.3, 127.9, 127.8, 127.5, 125.7, 124.9, 124.7, 124.4, 120.4, 55.4, 51.7.

**General procedure for the preparation of [ML^3^](ClO_4_)_2_ (M = Cu or Zn).** A solution of hydrated Cu(ClO_4_)_2_ or Zn(ClO_4_)_2_ (0.123 mmol) in acetonitrile (2 mL) was added to a solution of L^3^ (0.050 g, 0.123 mmol) in the same solvent (5 mL). The mixture was stirred at room temperature for 16 h and the solvent was removed under reduced pressure. The solid was treated with diethyl ether (5 mL), filtered, and dried under vacuum.

**[CuL^3^](ClO_4_)_2_·2H_2_O** ([CuL^3^](ClO_4_)_2_). Dark green solid. Yield: 0.052 g, 59%. MS-ESI^+^, *m*/*z* (%BPI): [CuL^3^(ClO_4_)]^+^, 567.1 (16%); [Cu(L^3^-H)]^+^, 467.1 (8%); [CuL^3^]^2+^, 234.1 (100%). Elem. Anal. Calcd for C_21_H_24_BCl_2_CuN_5_O_11_·2H_2_O: C, 35.8; H, 4.0; N, 10.0%. Found: C, 35.7; H, 3.6; N, 10.1%. Λ_M_ (MeOH, 10^−3^ M, 25 °C): 170.2 cm^2^·Ω^−1^·mol^−1^ (2:1 electrolyte). IR: 3364 ν(N-H/O-H), 1613 ν(Ph/Py), 1556 ν(N-H), 1341 ν(C-BO_2_), 1052 ν_a_(Cl-O), 619 ν_a_(O-Cl-O) cm^−1^. UV/vis diffuse reflectance spectroscopy: 585 nm.

**[ZnL^3^](ClO_4_)_2_·H_2_O** ([ZnL^3^](ClO_4_)_2_). Light brown solid. Yield: 0.041 g, 49%. MS-ESI^+^, *m*/*z* (%BPI): [Zn(L^3^-H)(OMe)]^+^, 482.1 (2%); [ZnL^3^(OMe)]^2+^, 241.6 (100%). Elem. Anal. Calcd for C_21_H_24_BCl_2_N_5_O_11_Zn·H_2_O: C, 36.7; H, 3.8; N, 10.2%. Found: C, 36.5; H, 4.0; N: 9.8%. Λ_M_ (MeOH, 10^−3^ M, 25 °C): 167.2 cm^2^·Ω^−1^·mol^−1^ (2:1 electrolyte). IR: 3363 ν(N-H/O-H), 1612 ν(Ph/Py), 1565 ν(N-H), 1341 ν(C-BO_2_), 1054 ν_a_(Cl-O), 619 ν_a_(O-Cl-O) cm^−1^. *δ*_H_ (solvent dmso-*d*_6_, 300 MHz, 298 K): 8.60 (m), 8.52 (s), 8.08 (m), 7.95 (s), 7.62–7.49 (m, 6H), 7.37 (m, 1H), 7.20 (m, 1H), 6.24 (s, 1H), 4.37 (d, H6A, ^2^*J* = 16.0 Hz), 4.05 (d, H6B, ^2^*J* = 16.0 Hz), 3.60 (m), 2.83 (m). *δ*_C_ (solvent dmso-*d*_6_, 75.5 MHz, 298 K): 155.7, 154.6, 147.6, 140.6, 139.2, 134.8, 127.8, 127.5, 124.7, 124.5, 124.3, 120.3, 57.2, 54.9, 34.7.

**General procedure for the preparation of [ML^4^](ClO_4_)_2_ (M = Cu or Zn) and [CuL^4^(SO_4_)].** A solution of hydrated Cu(ClO_4_)_2_, Zn(ClO_4_)_2_ or Cu(SO_4_) (0.123 mmol) in methanol (2 mL) was added to a solution of **L^4^** (0.050 g, 0.123 mmol) in the same solvent (5 mL). The mixture was stirred at room temperature for 16 h, and the precipitate formed was isolated by filtration, washed with methanol (2 mL) and diethyl ether (2 mL), and dried under vacuum. For the Zn^2+^ complex no precipitate was obtained, so the solvent was evaporated under reduced pressure and chloroform (5 mL) was added. The precipitate formed was stirred at room temperature for 16 h, filtered, washed with 5 mL of chloroform and 5 mL of diethyl ether, and dried under vacuum.

**[CuL^4^](ClO_4_)_2_·2H_2_O** ([CuL^4^](ClO_4_)_2_). Light blue solid. Yield: 0.075 g, 86%. MS-ESI^+^, *m*/*z* (%BPI): [CuL^4^(ClO_4_)]^+^, 568.0 (83%); [Cu(L^4^-H)]^+^, 468.1 (22%); [CuL^4^]^2+^, 234.6 (100%). Elem. Anal. Calcd for C_21_H_22_Cl_2_CuN_6_O_11_·2H_2_O: C, 35.8; H, 3.7; N, 11.9%. Found: C, 35.9; H, 3.4; N, 11.5%. Λ_M_ (MeOH, 10^−3^ M, 25 °C): 144.5 cm^2^·Ω^−1^·mol^−1^. IR: 3584, 3410, 3342 ν(N-H), 3089–2780 ν(C-H), 1697 ν(C=O), 1613, 1597 ν(Ph/Py), 1550 ν(N-H), 1504 ν_a_(NO_2_), 1334 ν_s_(NO_2_), 1093, 1061 ν_a_(Cl-O), 620 ν_a_(O-Cl-O) cm^−1^. UV/vis diffuse reflectance spectroscopy: 633 nm.

**[CuL^4^](SO_4_)·1.5H_2_O** ([CuL^4^(SO_4_)]). Light blue solid. Yield: 0.047 g, 64%. MS-ESI^+^, *m*/*z* (%BPI): [Cu(L^4^+H)(SO_4_)]^+^, 566.1 (20%); [Cu(L^4^-H)]^+^, 468.1 (3%); [CuL^4^]^2+^, 234.6 (52%). Elem. Anal. Calcd for C_21_H_22_CuN_6_O_7_S·1.5H_2_O: C, 42.5; H, 4.3; N, 14.2%. Found: C, 42.7; H, 3.9; N, 13.9%. Λ_M_ (MeOH, 10^−3^ M, 25 °C): the low solubility of this complex in methanol has prevented us from determining the conductivity value for this compound. IR: 3254 ν(N-H), 3117–2943 ν(C-H), 1692 ν(C=O), 1612, 1595 ν(Ph/Py), 1562 ν(N-H), 1502 ν_a_(NO_2_), 1324 ν_s_(NO_2_), 1106, 1049, 1029 ν_a_(S-O), 600, 584 ν_a_(O-S-O) cm^−1^. UV/vis diffuse reflectance spectroscopy: 706 nm. Slow evaporation of a solution of the complex in a dimethylformamide/water mixture provided dark blue single crystals suitable for X-ray diffraction analysis.

**[ZnL^4^](ClO_4_)_2_·CHCl_3_** ([ZnL^4^](ClO_4_)_2_). Light yellow solid. Yield 0.092 g, 95%. MS-ESI^+^, *m*/*z* (%BPI): [ZnL^4^(ClO_4_)]^+^, 569.1 (74%); [Zn(L^4^-H)]^+^, 469.1 (100%); [ZnL^4^]^2+^, 235.1 (83%). Elem. Anal. Calcd for C_21_H_22_Cl_2_N_6_O_11_Zn·CHCl_3_: C, 33.4; H, 2.9; N, 10.6%. Found: C, 33.8; H, 3.1; N: 10.7%. Λ_M_ (MeOH, 10^−3^ M, 25 °C): 161.9 cm^2^·Ω^−1^·mol^−1^ (2:1 electrolyte). IR: 3372 ν(N-H), 1647 ν(C=O), 1614 ν(Ph/Py), 1556 ν(N-H), 1506 ν_a_(NO_2_), 1329 ν_s_(NO_2_), 1094, 1051 ν_a_(Cl-O), 619 ν_a_(O-Cl-O) cm^−1^. *δ*_H_ (solvent dmso-*d*_6_, 300 MHz, 298 K): 9.39 (s, 1H, H11), 8.61 (m, 2H), 8.16–8.06 (m, 4H), 7.63–7.57 (m, 6H), 6.52 (m, 1H, H9), 4.38 (d, 2H, H6A, ^2^*J* = 16.0 Hz), 4.06 (d, 2H, H6B, ^2^*J* = 16.0 Hz), 3.45 (m), 2.82 (m, 2H). *δ*_C_ (solvent dmso-*d*_6_, 75.5 MHz, 298 K): 154.6, 154.5, 147.5, 146.9, 140.6, 125.1, 124.7, 124.4, 117.0, 56.8, 54.0, 34.5.

**Procedure for the preparation of [CuL^5^](ClO_4_)_2_.** A solution of hydrated Cu(ClO_4_)_2_ (0.150 mmol) in acetonitrile (2 mL) was added to a solution of L^5^ (0.050 g, 0.150 mmol) in acetonitrile (5 mL). The mixture was stirred at room temperature for 16 h and the solvent was removed under reduced pressure. The solid was treated with diethyl ether (5 mL), filtrated and dried under vacuum.

**[CuL^5^](ClO_4_)_2_·H_2_O** ([CuL^5^](ClO_4_)_2_). Dark blue solid. Yield: 0.075 g, 82%. MS-ESI^+^, *m*/*z* (%BPI): [CuL^5^(ClO_4_)]^+^, 495.1 (70%); [Cu(L^5^-H)]^+^, 395.1 (100%). Elem. Anal. Calcd for C_19_H_20_BCl_2_CuN_3_O_10_·H_2_O: C, 37.2; H, 3.6; N, 6.9%. Found: C, 37.4; H, 3.8; N, 7.1%. Λ_M_ (MeOH, 10^−3^ M, 25 °C): 171.3 cm^2^·Ω^−1^·mol^−1^ (2:1 electrolyte). IR: 3441 ν(O-H), 1613, 1576, 1487 ν(Ph/Py), 1346 ν(C-BO_2_), 1048 ν_a_(Cl-O), 619 ν_a_(O-Cl-O) cm^−1^. UV/vis diffuse reflectance spectroscopy: 603 nm.

### 3.3. X-ray Diffraction Studies

Crystals of [CuL^4^(SO_4_)] were obtained by evaporation of a solution of the isolated complex, as described above. Crystals of [CuL^4^(H_2_PPi)] were grown by slow evaporation of an acetonitrile–methanol (1:1) mixture containing stoichiometric amounts of [CuL^4^](ClO_4_)_2_ and sodium pyrophosphate. Finally, [{CuL^4^}_2_(μ-PPi)] and [{ZnL^4^}_2_(μ-PPi)] were crystallized by slow evaporation of solutions of [CuL^4^](ClO_4_)_2_ (acetonitrile-methanol mixture) or [ZnL^4^](ClO_4_)_2_ (deuterium oxide) in the presence of 0.5 equiv. of sodium pyrophosphate.

Three-dimensional X-ray data were collected on Bruker X8 APEXII CCD ([{ZnL^4^}_2_(µ-PPi)], [{CuL^4^}_2_(µ-PPi)] and [CuL^4^(H_2_PPi)]) or BRUKER-NONIUS X8 APEX CCD ([CuL^4^(SO_4_)]) diffractometers. The data of [{CuL^4^}_2_(µ-PPi)], [CuL^4^(H_2_PPi)]) and [CuL^4^(SO_4_)] were corrected for Lorentz and polarization effects and for absorption by semiempirical methods based on symmetry-equivalent reflections [79]. The crystal selected for [{ZnL^4^}_2_(µ-PPi)] was a twin, so that the correction for absorption was made with TWINABS [80]. Two domains were found and two corrected hkl files were generated, one for solving the structure (HKLF 4) and another for the final refinement (HKLF 5). Complex scattering factors were taken from the program SHELX-2017 running under the WinGX program system as implemented on a Pentium^®^ computer [81,82]. The structures were solved by using the charge-flipping method with Superflip [83] (for complex [{ZnL^4^}_2_(µ-PPi)] or Patterson methods with DIRDIF2008 [84] (for [{CuL^4^}_2_(µ-PPi)], [CuL^4^(H_2_PPi)] and [CuL^4^(SO_4_)]). All of the structures were refined by full-matrix least-squares on *F*^2^ (SHELXL-2017) [85]. Hydrogen atoms were included in calculated positions and refined in riding mode for all of the compounds with the following exceptions: hydrogen atoms of urea NH moieties were refined freely in the final stages of refinement and hydrogen atoms of water molecules (one molecule per unit cell in [CuL^4^(H_2_PPi)] and six molecules per unit cell in crystals of [{ZnL^4^}_2_(µ-PPi)]) were located in a difference electron-density map and the H⋯H and O-H distances restrained.

In the final steps of refinement of the crystal structure of [{ZnL^4^}_2_(µ-PPi)], the data were cleaned up with SQUEEZE [86] to avoid the problems generated by disordered methanol and acetonitrile molecules in two voids with volumes of 255 and 115 Å^3^ and electron counts of 82 and 35, respectively, which agree with the presence of two acetonitrile and one methanol molecule, respectively.

Finally, refinement converged with anisotropic displacement parameters for all non-hydrogen atoms for all four structures. Crystal data and details on data collection and refinement are summarized in Table 5.

### 3.4. Computational Details

All of the calculations presented in this work were performed employing the Gaussian 09 package (Revision B.01) [87]. Full geometry optimizations of [ZnL^4^(H_2_PPi)] and [{ZnL^4^}_2_(μ-PPi)] complexes were carried out in dmso solution employing DFT within the hybrid meta-GGA approximation with the TPSSh exchange-correlation functional [88]. Input geometries were generated from the crystallographic data of [CuL^4^(H_2_PPi)] and [{ZnL^4^}_2_(μ-PPi)] by replacing the Cu(II) metal ion in the first one. For geometry optimization purposes we used the standard Ahlrichs’ valence double-ξ basis set including polarization functions (SVP) [89]. No symmetry constraints have been imposed during the optimizations. The stationary points found on the potential energy surfaces as a result of geometry optimizations were tested to represent energy minima rather than saddle points via frequency analysis. The default values for the integration grid (75 radial shells and 302 angular points) and the SCF energy convergence criteria (10^−8^ a.u.) were used in all the calculations. The ^31^P NMR shielding tensors were calculated using the GIAO [90] method at the TPSSh/TZVP [91] level. The isotropic shielding constants (σ) were converted to chemical shifts with respect to 85% aqueous H_3_PO_4_ by using the following expression [92,93]:

δ^calc^ = σ^calc^(PH_3_) − σ^calc^(complex) − 266.1 (1)

The shielding tensors of PH_3_ were calculated at the same computational level.

Throughout this work solvent effects (dmso) were included by using the polarizable continuum model (PCM), in which the solute cavity is built as an envelope of spheres centered on atoms or atomic groups with appropriate radii. In particular, we used the integral equation formalism (IEFPCM) variant as implemented in Gaussian 09 [94].

## 4. Conclusions

We have presented a series of Cu^2+^ and Zn^2+^ complexes containing dipicolylamine units for metal ion coordination and urea groups. In the absence of phosphorylated anions, the urea group coordinates to the metal ion through one of the nitrogen atoms. Addition of monophosphate anions does not provoke the decoordination of the urea group, while the addition of PPi, ADP, or ATP causes decoordination of the urea group and a change of the coordination environment from distorted octahedral to trigonal bipyramidal. The coordination of the PPi and ADP anions to the metal ion appears to be reinforced by the presence of hydrogen bonds with the urea unit, as also observed in the solid state, while these interactions seem to be weaker in the case of ATP. The [CuL^4^]^2+^ and [CuL^5^]^2+^ complexes show important selectivities towards PPi, ADP, and ATP over anions containing a single phosphate group. The results indicate that the cooperative binding is established for the complexes derived from L^4^ during the anion recognition and that it is necessary to consider the N-type coordination mode of the urea moiety to prevent the receptor hydrolysis when designing urea based receptors with different delocalized withdrawing groups in their backbones.

## Figures and Tables

**Table 1:** Bond distances (Å) of the metal coordination environments.

	[CuL^4^(SO_4_)]	[CuL^4^(H_2_PPi)]	[{CuL^4^}_2_(μ-PPi)]	[{ZnL^4^}_2_(μ-PPi)]
M(1)-N(3)	2.728(3)	2.670(1)	-	-
M(1)-N(4)	2.044(3)	2.039(1)	2.100(2)	2.340(6)
M(1)-N(5)	2.005(3)	1.994(1)	1.990(2)	2.045(7)
M(1)-N(6)	2.000(2)	1.991(1)	1.989(2)	2.041(6)
M(1)-O(4)	1.960(2)	1.930(1)	1.938(1)	2.021(5)
M(1)-O(5)	2.247(2)	-		-
M(1)-O(8)	-	2.263(1)	2.172(2)	1.949(5)

**Table 2:** Intramolecular hydrogen-bonding interactions observed in the solid state structures of [CuL^4^(SO_4_)], [{CuL^4^}_2_(μ-PPi)] and [{ZnL^4^}_2_(μ-PPi)].

		d(D-H)/Å	d(H⋯A)/Å	d(D⋯A)/Å	D-H⋯A/°
[CuL^4^(SO_4_)]	N(2)-H(2N)⋯O(6)	0.82(3)	1.97(3)	2.768(3)	161(3)
[{CuL^4^}_2_(μ-PPi)]	N(2)-H(2N)⋯O(10)	0.79(2)	2.04(3)	2.830(3)	175(2)
	N(3)-H(3N)⋯O(8)	0.74(3)	2.08(3)	2.797(2)	161(3)
	N(8)-H(8N)⋯O(6)	0.79(2)	1.98(3)	2.765(2)	173(2)
	N(9)-H(9N)⋯O(5)	0.82(3)	1.94(3)	2.751(2)	169(2)
[{ZnL^4^}_2_(μ-PPi)]	N(2)-H(2N)⋯O(10)	0.73(9)	2.12(9)	2.850(9)	172(10)
	N(3)-H(3N)⋯O(8)	0.76(9)	2.13(9)	2.880(9)	168(10)
	N(8)-H(8N)⋯O(6)	0.82(9)	2.05(9)	2.870(8)	178(9)
	N(9)-H(9N)⋯O(5)	0.75(9)	2.15(9)	2.876(9)	163(10)

**Table 3:** Association constants (log *K* values) and p*A* values obtained from spectrophotometric titrations in aqueous solutions (pH 7, MOPS buffer). ^1^

		Phosphate	AMP	CMP	UMP	PPi	ADP	ATP
[CuL^3^]^2+^	Log *K*_11_	4.20(1)	4.35(1)	3.59(1)	4.02(1)	4.82(4)	4.02(1)	4.28(1)
	Log *K*_21_	-	-	-	-	4.14(5)	2.42(4)	3.00(4)
	p*A*	5.16	5.31	4.56	4.98	7.77	5.44	6.15
[CuL^4^]^2+^	Log *K*_11_	3.44(1)	3.80(1)	3.31(1)	^2^	^2^	6.83(9)	6.26(5)
	Log *K*_21_	1.72(7)	-	-			4.8(1)	4.32(6)
	p*A*	4.56	4.76	4.29			10.44	9.39
[CuL^5^]^2+^	Log *K*_11_	3.45(3)	3.31(3)	3.51(1)	3.00(1)	6.1(3)	4.89(2)	4.61(1)
	Log *K*_21_	3.67(7)	-	-	-	-	3.81(2)	3.41(3)
	p*A*	5.94	4.29	4.48	4.00	11.01	7.52	6.85

^1^ p*A* values defined as p*A*= −log[Anion]_free_, with [Anion] = 0.001 M and [(CuL^n^)^2+^] = 0.01 M (pH 7.0). ^2^ Precipitation during the titration prevented the determination of association constants.

**Table 4:** ^31^P measured chemical shifts (dmso-*d*_6_) and calculated chemical shifts using the GIAO method at the TPSSh/TZVP level (see Computational Details) for [ZnL^4^(H_2_PPi)] and [{ZnL^4^}_2_(μ-PPi)] species (fully deprotonated and doubly protonated forms of the pyrophosphate anion were considered).

Species	δ (Experimental)	δ (Calculated) Deprotonated Form	δ (Calculated) Doubly Protonated Form
[{ZnL^4^}_2_(μ-PPi)]	−5.00	−5.90	−10.37
[ZnL^4^(H_2_PPi)]	−7.75	−72.15	−6.74

**Table 5:** Crystal data and refinement details.

	[CuL^4^(SO_4_)]	[CuL^4^(H_2_PPi)]	[{CuL^4^}_2_(μ-PPi)]	[{ZnL^4^}_2_(μ-PPi)]
formula	C_21_H_22_CuN_6_O_7_S	C_23_H_29_CuN_7_O_11_P_2_	C_42_H_44_Cu_2_N_12_O_13_P_2_	C_42_H_56_N_12_O_19_P_2_Zn
MW	566.04	705.01	1113.91	1225.66
crystal system	orthorhombic	triclinic	triclinic	monoclinic
space group	*Pbca*	*P* −1	*P* −1	*P* 2_1_/*n*
T/K	100(2)	100(2)	100(2)	100(2)
a/Å	19.636(3)	7.606(2)	13.5195(9)	10.2371(6)
b/Å	8.913(1)	11.984(3)	13.6036(9)	17.6978(10)
c/Å	25.692(3)	17.360(4)	14.5281(10)	28.5263(16)
α/deg	90	106.18(1)	76.346(4)	90
β/deg	90	95.12(1)	79.212(5)	95.104(3)
γ/deg	90	105.26(1)	76.738(4)	90
V/Å^3^	4496.30(10)	1443.5(6)	2502.0(3)	5147.7(5)
F(000)	2328	726	1144	2536
Z	8	2	2	4
λ, Å (Mo Kα)	0.71073	0.71073	0.71073	0.71073
D_calc_/g·cm^−3^	1.672	1.622	1.479	1.581
μ/mm^−1^	1.123	0.939	0.987	1.081
*θ* range/deg	2.969–25.149	1.855–30.706	1.457–26.389	1.356–26.391
R_int_	0.0943	0.0424	0.0391	0.1223 ^c^
reflns obsd	4016	8880	10203	13103
GOF on *F*^2^	1.051	1.013	1.023	1.011
R1 ^a^	0.038	0.0300	0.0320	0.0768
*w*R2 (all data) ^b^	0.0980	0.0746	0.0765	0.1789
Largest diff. peak and hole/e Å^−3^	0.464 and −0.533	0.532 and −0.366	0.397 and −0.434	0.959 and −1.946

^a^ R_1_ = Σ||F_o_| − |F_c_||/Σ|F_o_|. ^b^ wR_2_ (all data) = {Σ[w(||F_o_|^2^ − |F_c_|^2^)^2^]/Σ[w(F_o_^4^)]}^1/2^. ^c^ R_int_ is based on agreement between observed single and composite intensities and those calculated from refined unique intensities and twin fractions for all 64,393 observations.

## Supplementary Materials

Supplementary materials are available online.
