# Supplementary material for: Recognition of AMP, ADP and ATP through Cooperative Binding by Cu(II) and Zn(II) Complexes Containing Urea and/or Phenylboronic Acid Moieties
Source: Molecules. 2018 Feb 22;23(2):479. doi: 10.3390/molecules23020479 (PMC6017333; doi:10.3390/molecules23020479)
Supplement: Supplementary file 1 [file molecules-23-00479-s001.pdf]

Supplementary Materials

Article

Recognition of AMP, ADP and ATP through cooperative binding by Cu(II) and Zn(II) complexes containing urea and/or phenylboronic acid moieties

Israel Carreira-Barral [1,2], Isabel Fernández-Pérez [1], Marta Mato-Iglesias [1], Andrés de Blas [1], Carlos Platas-Iglesias [1] and David Esteban-Gómez [1,*]

1 Universidade da Coruña, Centro de Investigacións Científicas Avanzadas (CICA) and Departamento de Química, Facultade de Ciencias, 15071, A Coruña, Galicia, Spain. sabela@udc.es (I.F.-P.); mmato@udc.es (M.M.-I.); andres.blas@udc.es (A.B.); carlos.platas.iglesias@udc.es (C.P.-I.)

2 Universidad de Burgos, Facultad de Ciencias, Departamento de Química, 09001, Burgos, Spain; icarreira@ubu.es (I.C.-B.)

* Correspondence: david.esteban@udc.es; Tel.: +34-881-01-5597

Academic Editor: name
Received: date; Accepted: date; Published: date

	Contents	Page
**Figure S1**	View of the {[CuL^4(SO$_4$)]}$_n$ coordination polymer present in crystals of [CuL^4(SO$_4$)]	**S3**
**Figure S2**	Coordination polyhedron observed for Cu(II) ion in the X-ray crystal structures of [CuL^4(SO$_4$)] (left) and [CuL^4(H$_2$PPi)] (right)	**S3**
**Figure S3**	^{1}H NMR spectra of the [ZnL^1](ClO$_4$)$_2$ complex recorded upon 4 h in wet dmso-*d*$_6$ (300 MHz, 298 K)	**S4**
**Figure S4**	^{1}H NMR spectra of the [ZnL^2](ClO$_4$)$_2$ complex recorded as a function of time in wet dmso-*d*$_6$ (300 MHz, 298 K)	**S4**
**Figure S5**	Hydrolysis rates (k_{obs}) determined by UV/vis spectroscopy following the absorption bands of 3-nitroaniline (390 nm) for [CuL^2]$^{2+}$ and 4-nitroaniline (430 nm) for [CuL^4]$^{2+}$ complexes: (a) [CuL^2]$^{2+}$ at 25 °C (10^{-4} M in H$_2$O, pH 5.5 and 7.0, MOPS 0.1 M); (b) [CuL^2]$^{2+}$ at 50 °C (10^{-4} M in H$_2$O, pH 5.5 and 7.0, MOPS 0.1 M); (c) [CuL^4]$^{2+}$ at 25 °C (5·10^{-4} M in H$_2$O, pH 5.5 and 7.0, MOPS 0.1 M) and (d) [CuL^4]$^{2+}$ at 50 °C (5·10^{-4} M in H$_2$O, pH 5.5 and 7.0, MOPS 0.1 M)	**S5**
**Figure S6**	Family of UV/vis spectra taken during the course of the titration of [CuL^4]$^{2+}$ (5 × 10^{-3} M in H$_2$O, pH 7.0, MOPS 0.1 M, 25 °C) with a standard solution (0.5 M) of Na$_2$CMP. Inset: titration profile at selected wavelength *vs* equivalents of anion and species distribution diagram	**S5**
**Figure S7**	Family of UV/vis spectra taken during the course of the titration of [CuL^3]$^{2+}$ (10^{-3} M in H$_2$O, pH 7.0, MOPS 0.1 M, 25 °C) with standard solutions (0.1 M) of: (a) NaH$_2$PO$_4$; (b) Na$_2$AMP; (c) Na$_2$CMP; (d) Na$_2$UMP; (e) Na$_2$PPi; (f) Na$_2$ADP; (g) Na$_2$ATP. Insets: titration profile at selected wavelengths *vs* equivalents of anion and species distribution diagram	**S6**

**Figure S8**	Family of UV/vis spectra taken during the course of the titration of $[CuL^5]^{2+}$ (10^{-3} M in H_2O, pH 7.0, MOPS 0.1 M, 25 °C) with standard solutions (0.1 M) of: (a) NaH_2PO_4; (b) Na_2AMP; (c) Na_2CMP; (d) Na_2UMP; (e) Na_2PPi; (f) Na_2ADP; (g) Na_2ATP. Insets: titration profile at selected wavelengths *vs* equivalents of anion and species distribution diagram	**S7**
**Figure S9**	Isotopic profiles obtained by high resolution mass spectra recorded by electrospray ionization (ESI^+) (on the left, aqueous solution, 1 equiv. of anion, pH 7.0) and calculated (on the right) for 1:1 entities ($[CuL^4]$:anion); $[Cu(L^4\text{-H}):PPi+2Na]^+$ (top), $[CuL^4:ADP+Na]^+$ (middle) and $[Cu(L^4\text{-H}):ATP+2Na]^+$ (bottom).	**S8**
**Figure S10**	Isotopic profiles obtained by high resolution mass spectra recorded by electrospray ionization (ESI^+) (on the left, dmso, 1 equiv. of nucleotide) and calculated (on the right) for 1:1 entities $[CuL^3:\text{nucleotide}+H]^+$; AMP (top), ADP (middle) and ATP (bottom)	**S9**
**Figure S11**	^1H NMR spectra (300 MHz, 298 K) recorded for $[ZnL^3]^{2+}$ complex in the presence of different anions as their sodium salts in dmso-d_6.	**S10**
**Figure S12**	^{31}P NMR spectra (500 MHz, 298 K) recorded for $[ZnL^3]^{2+}$ complex in the presence of AMP, ADP, ATP and PPi as their sodium salts in dmso-d_6. The ^{31}P NMR spectra of free metabolites (except for PPi due to its low solubility) are shown as colored traces.	**S10**
**Figure S13**	Geometries of $[\{ZnL^4\}_2(\mu\text{-PPi})]$ (left) and $[ZnL^4(H_2PPi)]$ (right) complexes obtained from DFT calculations (TPSSh/SVP level) in dmso solution.	**S11**
**Figure S14**	Labelling scheme of ligands L^1, L^3, L^4 and L^5 for ^1H and ^{13}C NMR signals assignment.	**S11**
**Table S1**	(ESI^+) Mass spectra data obtained for aqueous solutions of $[CuL^4](ClO_4)_2$ complex in the presence of one equivalent of PPi, ADP and ATP	**S7**
**Computational Data**	Cartesian coordinates (Å) of optimized geometries obtained with DFT calculations	**S12-S21**

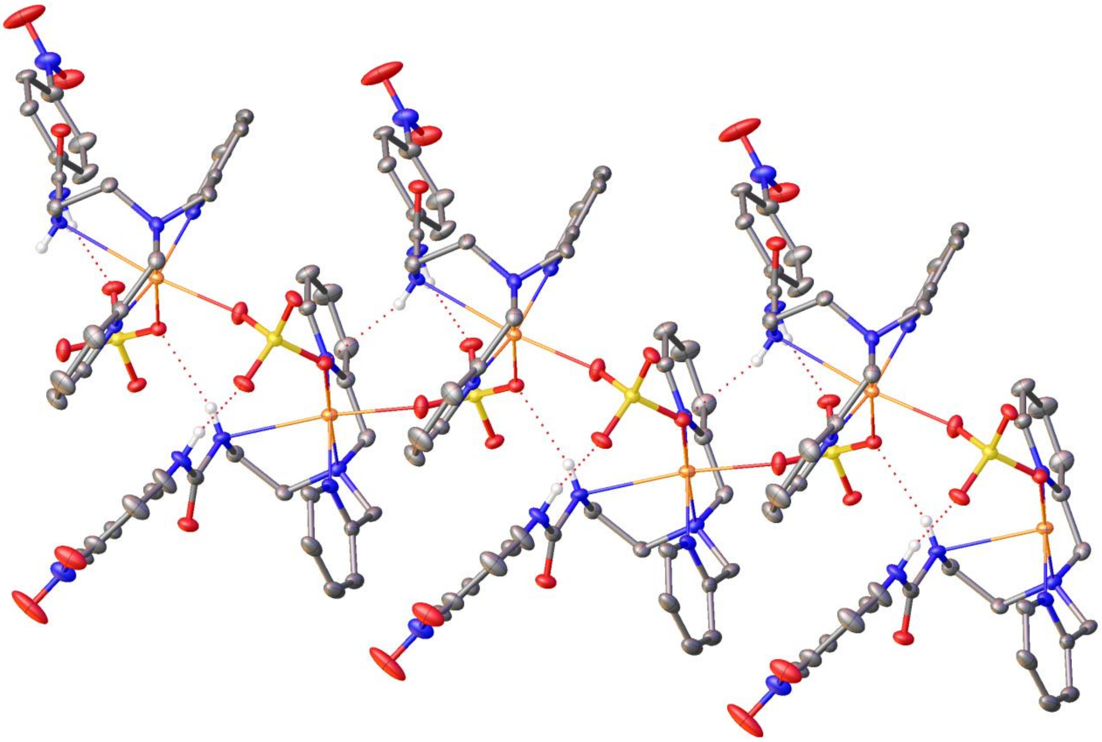

Figure S1. View of the {[CuL[4](SO_4)]}$_n$ coordination polymer present in crystals of [CuL[4](SO_4)].

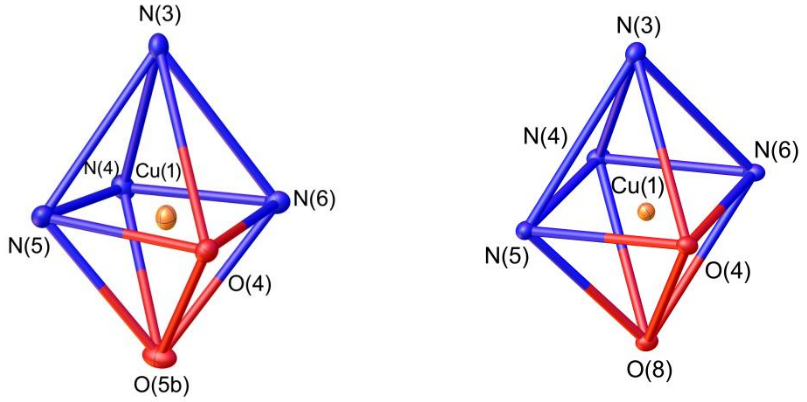

Figure S2. Coordination polyhedron observed for Cu(II) ion in the X-ray crystal structures of [CuL[4](SO_4)] (left) and [CuL[4](H_2PPi)] (right).

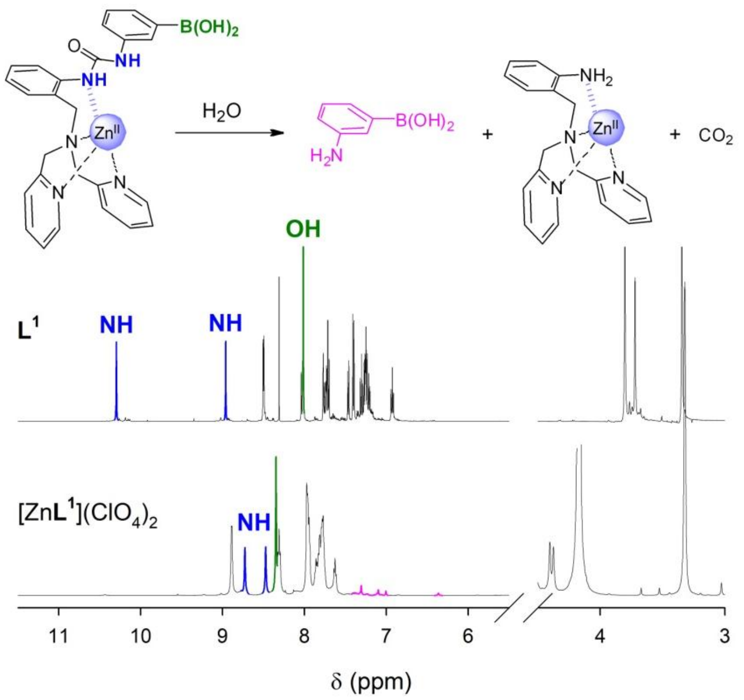

Figure S3. ^{1}H NMR spectra of the $[ZnL^{1}](ClO_4)_2$ complex recorded upon 4 h in wet dmso-d_6 (300 MHz, 298 K).

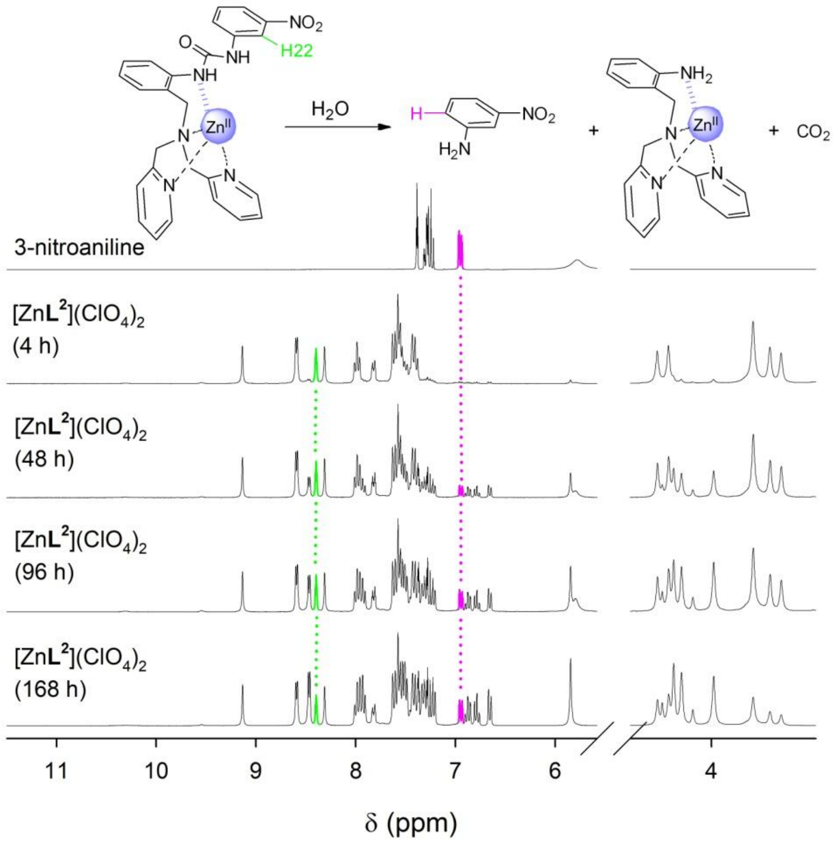

Figure S4. ^{1}H NMR spectra of the $[ZnL^{2}](ClO_4)_2$ complex recorded as a function of time in wet dmso-d_6 (300 MHz, 298 K).

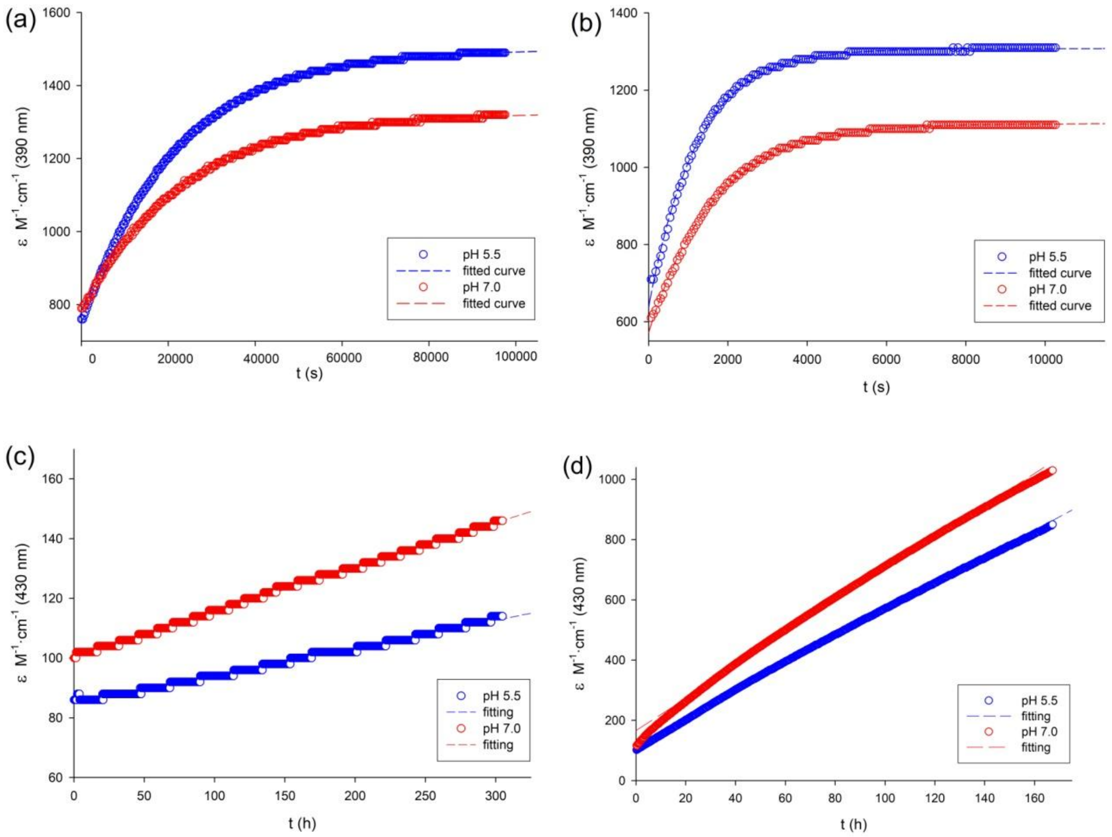

Figure S5. Hydrolysis rates (k_{obs}) determined by UV/vis spectroscopy following the absorption bands of 3-nitroaniline (390 nm) for $[CuL^2]^{2+}$ and 4-nitroaniline (430 nm) for $[CuL^4]^{2+}$ complexes: (a) $[CuL^2]^{2+}$ at 25 °C (10^{-4} M in H_2O, pH 5.5 and 7.0, MOPS 0.1 M); (b) $[CuL^2]^{2+}$ at 50 °C (10^{-4} M in H_2O, pH 5.5 and 7.0, MOPS 0.1 M); (c) $[CuL^4]^{2+}$ at 25 °C ($5\cdot10^{-4}$ M in H_2O, pH 5.5 and 7.0, MOPS 0.1 M) and (d) $[CuL^4]^{2+}$ at 50 °C ($5\cdot10^{-4}$ M in H_2O, pH 5.5 and 7.0, MOPS 0.1 M).

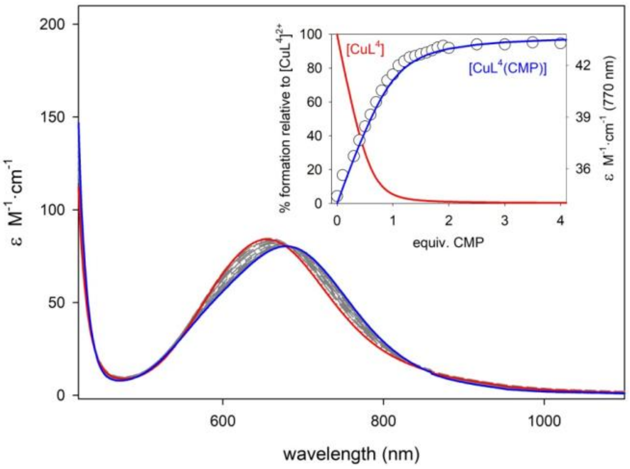

Figure S6. Family of UV/vis spectra taken during the course of the titration of $[CuL^4]^{2+}$ (5×10^{-3} M in H_2O, pH 7.0, MOPS 0.1 M, 25 °C) with standard solutions (0.5 M) of Na_2CMP. Inset: titration profile at selected wavelength *vs* equivalents of anion and species distribution diagram.

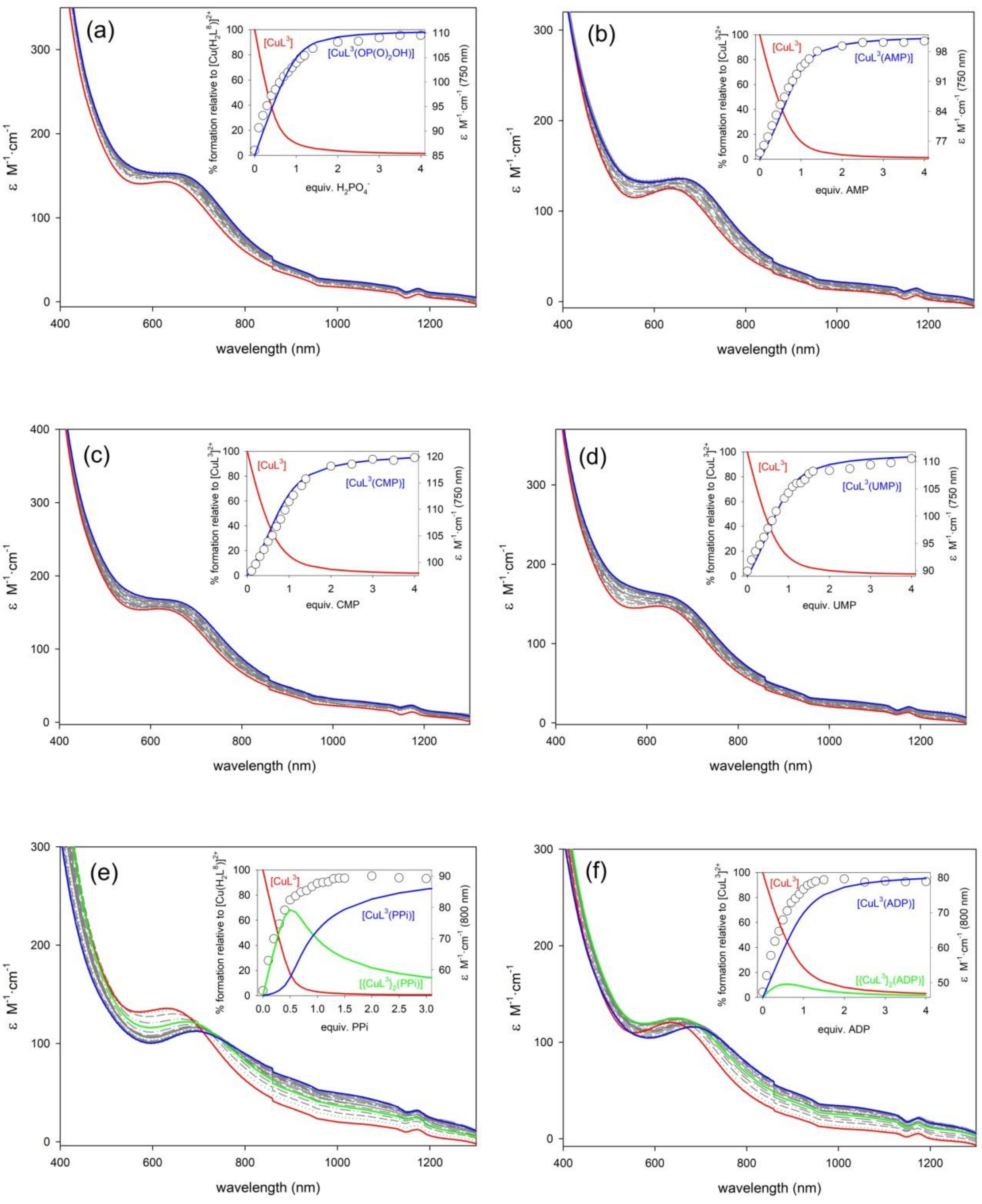

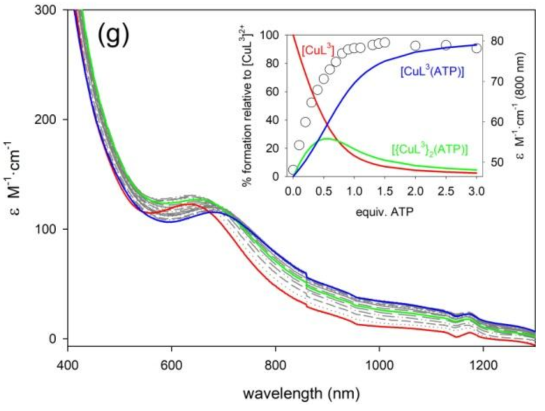

Figure S7. Family of UV/vis spectra taken during the course of the titration of $[CuL^3]^{2+}$ (10^{-3} M in H_2O, pH 7.0, MOPS 0.1 M, 25 °C) with standard solutions (0.1 M) of: (a) NaH_2PO_4; (b) Na_2AMP; (c) Na_2CMP; (d) Na_2UMP; (e) Na_2PPi; (f) Na_2ADP; (g) Na_2ATP. Insets: titration profile at selected wavelengths *vs* equivalents of anion and species distribution diagram.

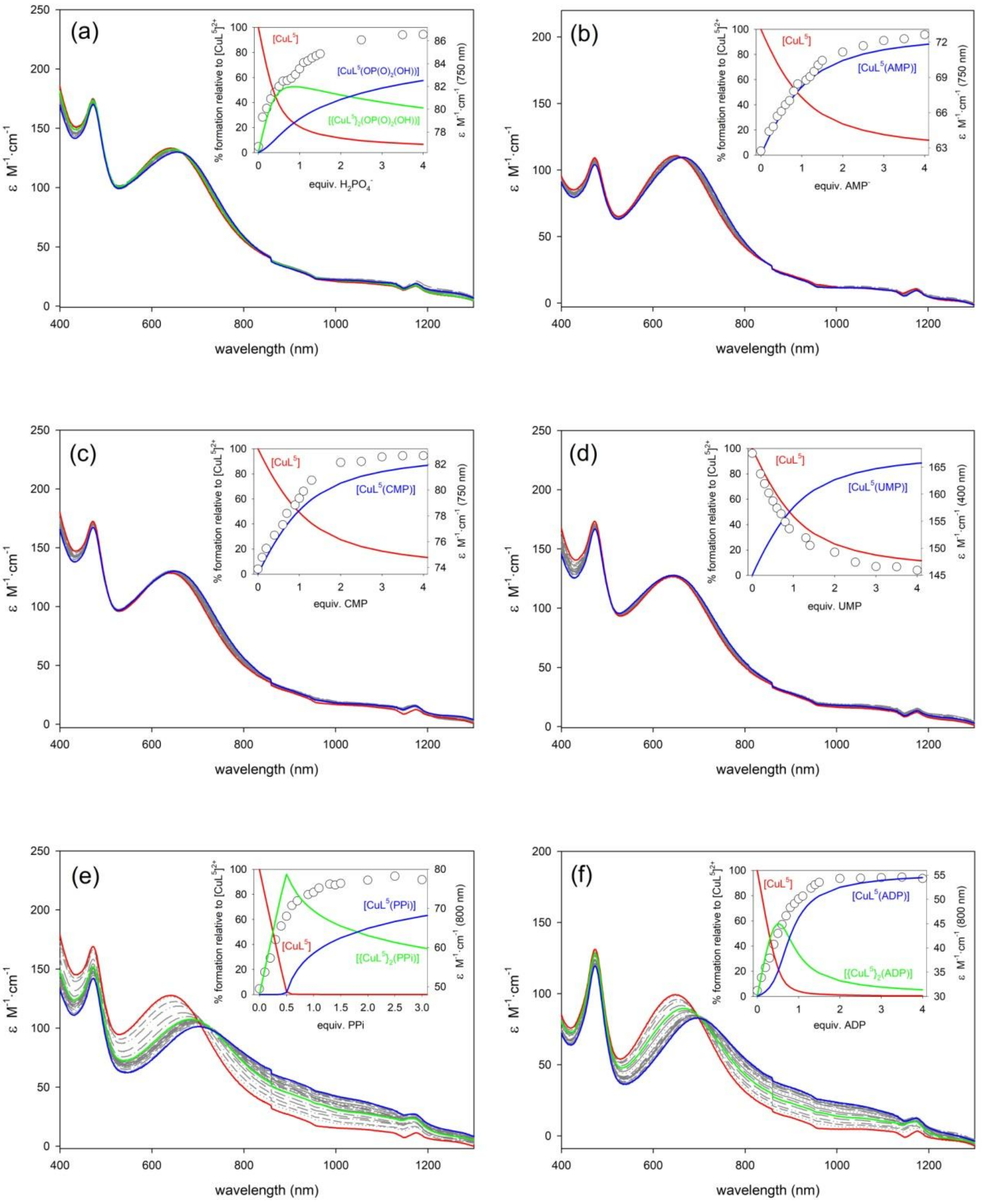

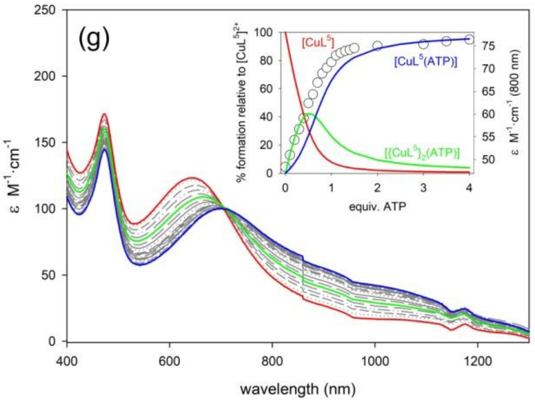

Figure S8. Family of UV/vis spectra taken during the course of the titration of $[CuL^5]^{2+}$ (10^{-3} M in H_2O, pH 7.0, MOPS 0.1 M, 25 °C) with standard solutions (0.1 M) of: (a) NaH_2PO_4; (b) Na_2AMP; (c) Na_2CMP; (d) Na_2UMP; (e) Na_2PPi; (f) Na_2ADP; (g) Na_2ATP. Insets: titration profile at selected wavelengths *vs* equivalents of anion and species distribution diagram.

Table S1. (ESI$^+$) Mass spectra data obtained for aqueous solutions of $[CuL^4](ClO_4)_2$ complex in the presence of one equivalent of PPi, ADP and ATP.

1:1 entities		2:1 entities	
$[CuL^4{:}H_2PPi{+}Na]^+$	668.0 (11%)	$[(CuL^4)_2{:}HPPi]^+$	1113.1 (10%)
$[CuL^4{:}HPPi{+}2Na]^+$	690.0 (22%)	$[(CuL^4)_2{:}PPi{+}Na]^+$	1135.1 (5%)
$[CuL^4{:}ADP{+}Na]^+$	917.1 (10%)		
$[CuL^4{:}ADP{+}2Na]^+$	939.1 (10%)	$[(CuL^4)_2{:}ADP]^+$	1364.2 (5%)
$[CuL^4{:}ATP{+}2Na]^+$	1019.0 (3%)	$[(CuL^4)_2{:}ATP{+}Na]^+$	1466.2 (2%)

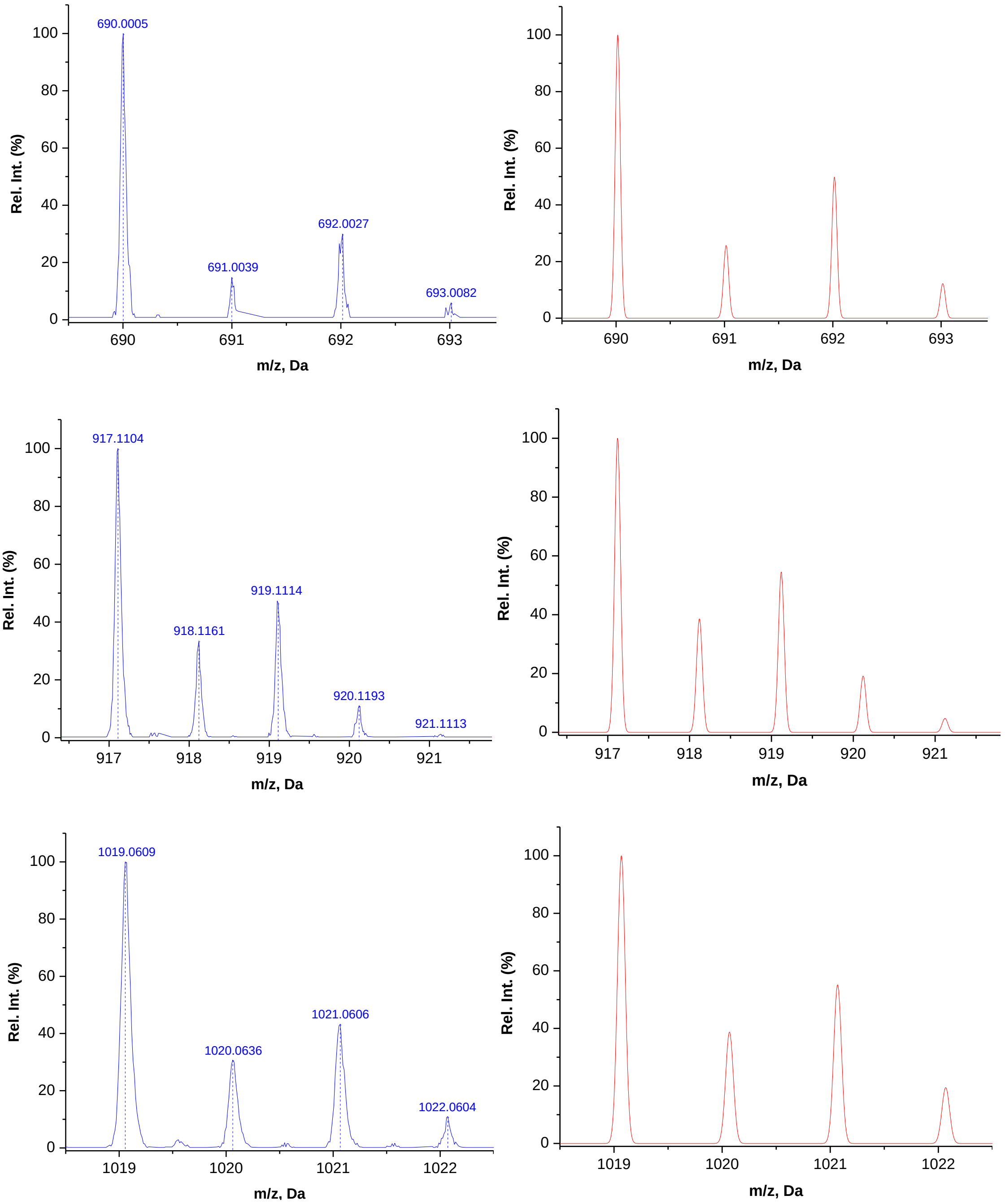

Figure S9. Isotopic profiles obtained by high resolution mass spectra recorded by electrospray ionization (ESI$^+$) (on the left, aqueous solution, 1 equiv. of anion, pH 7.0) and calculated (on the right) for 1:1 entities ($[CuL^4]$:anion); $[Cu(L^4{-}H){:}PPi{+}2Na]^+$ (top), $[CuL^4{:}ADP{+}Na]^+$ (middle) and $[Cu(L^4{-}H){:}ATP{+}2Na]^+$ (bottom).

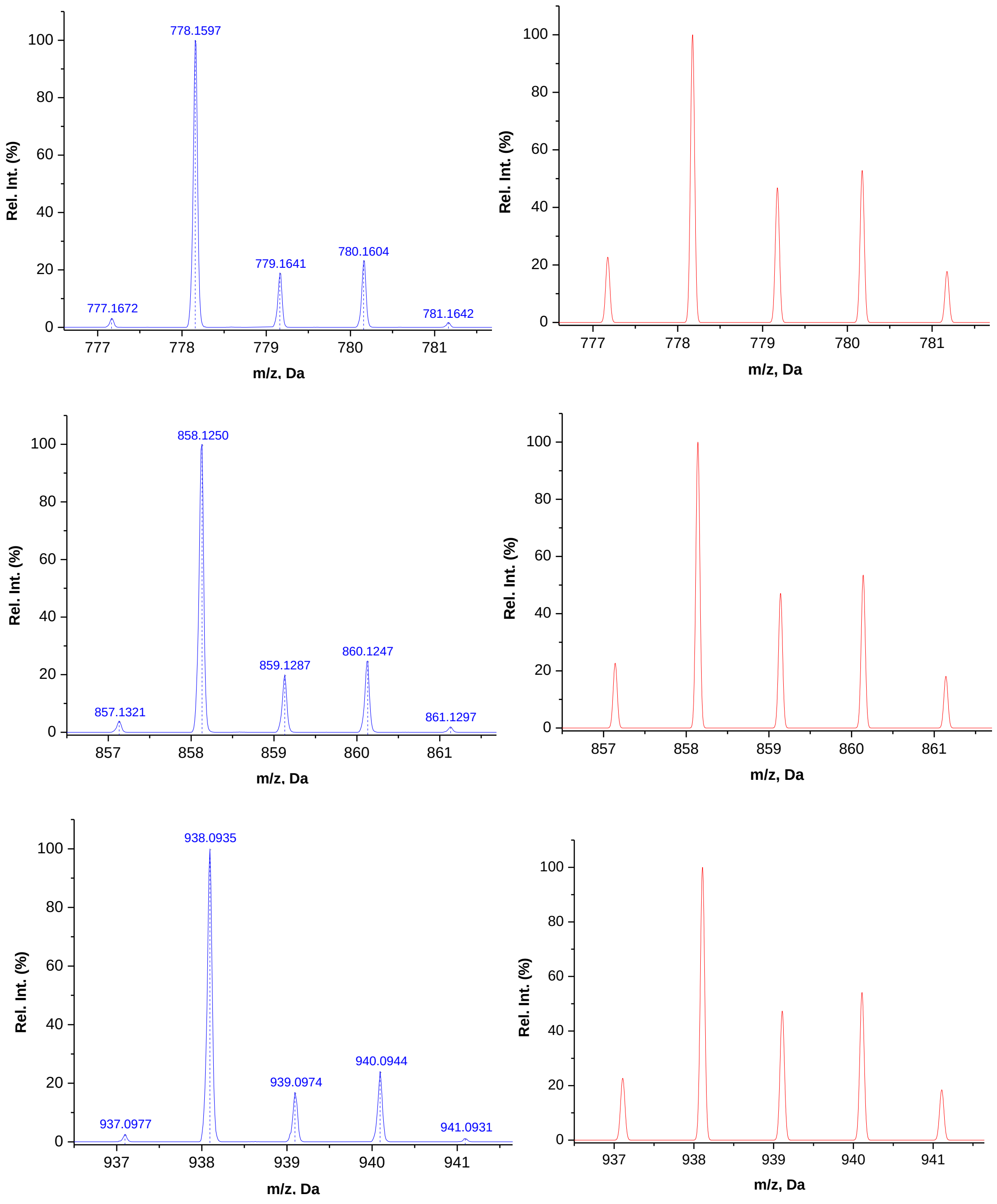

Figure S10. Isotopic profiles obtained by high resolution mass spectra recorded by electrospray ionization (ESI^+) (on the left, dmso, 1 equiv. of nucleotide) and calculated (on the right) for 1:1 entities [CuL^3:nucleotide+H]$^+$; AMP (top), ADP (middle) and ATP (bottom).

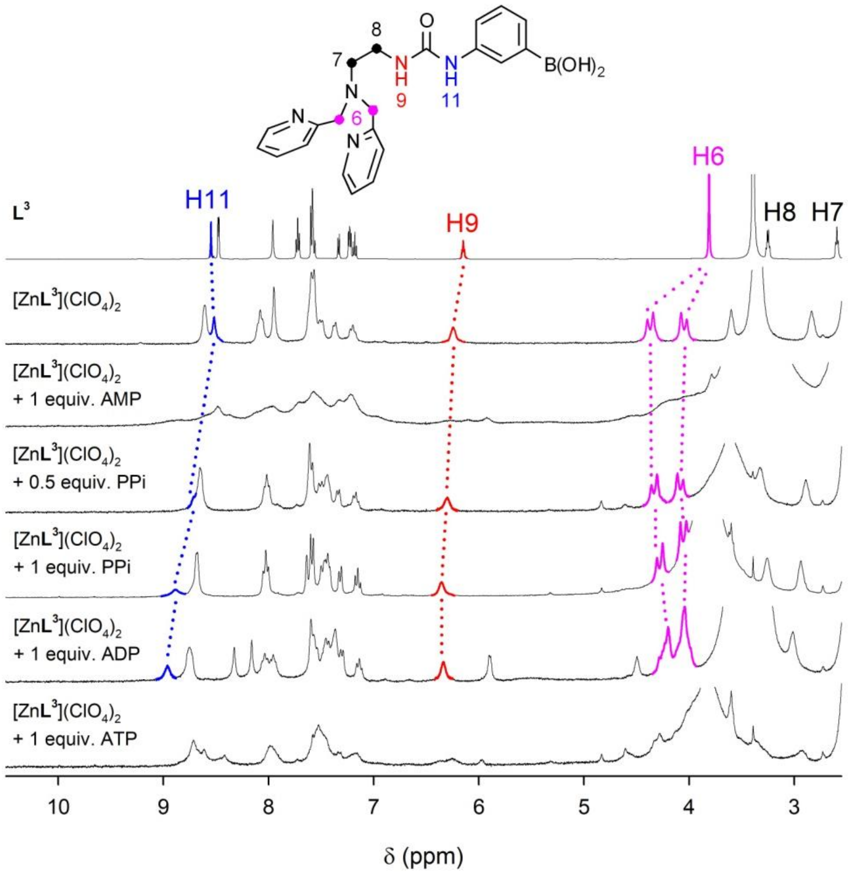

Figure S11. ^{1}H NMR spectra (300 MHz, 298 K) recorded for $[ZnL^3]^{2+}$ complex in the presence of different anions as their sodium salts in dmso-d_6.

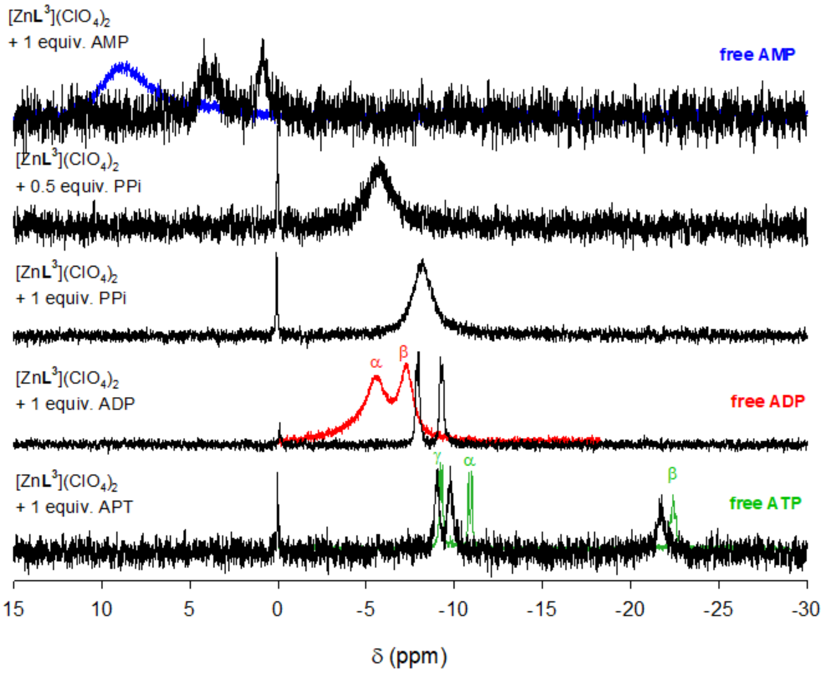

Figure S12. ^{31}P NMR spectra (500 MHz, 298 K) recorded for $[ZnL^3]^{2+}$ complex in the presence of AMP, ADP, ATP and PPi as their sodium salts in dmso-d_6. The ^{31}P NMR spectra of free metabolites (except for PPi due to its low solubility) are shown as colored traces.

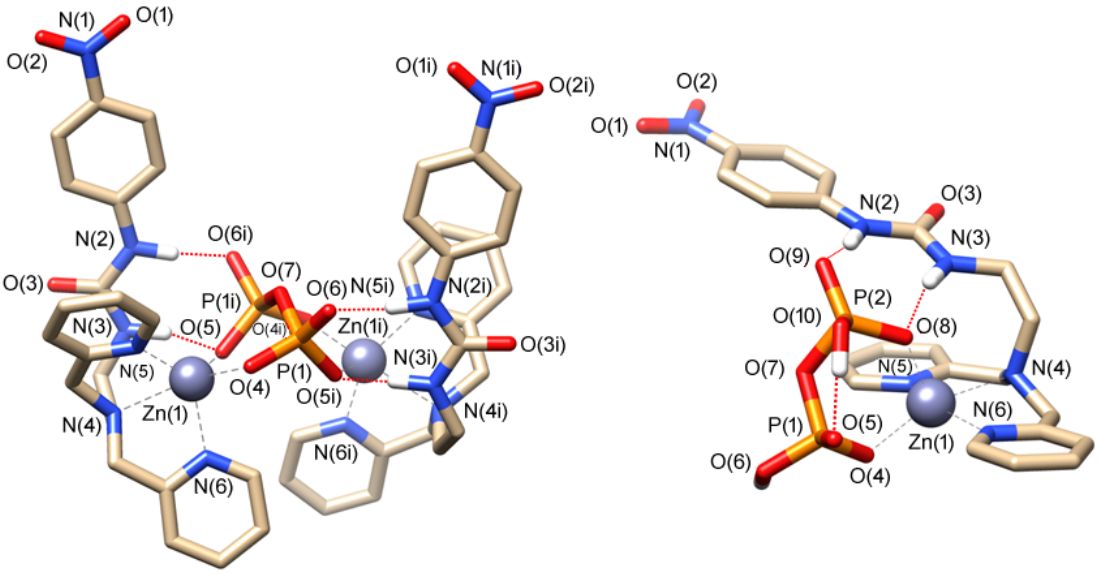

Figure S13. Geometries of [{ZnL⁴}₂(μ-PPi)] (left) and [ZnL⁴(H₂PPi)] (right) complexes obtained from DFT calculations (TPSSh/SVP level) in dmso solution.

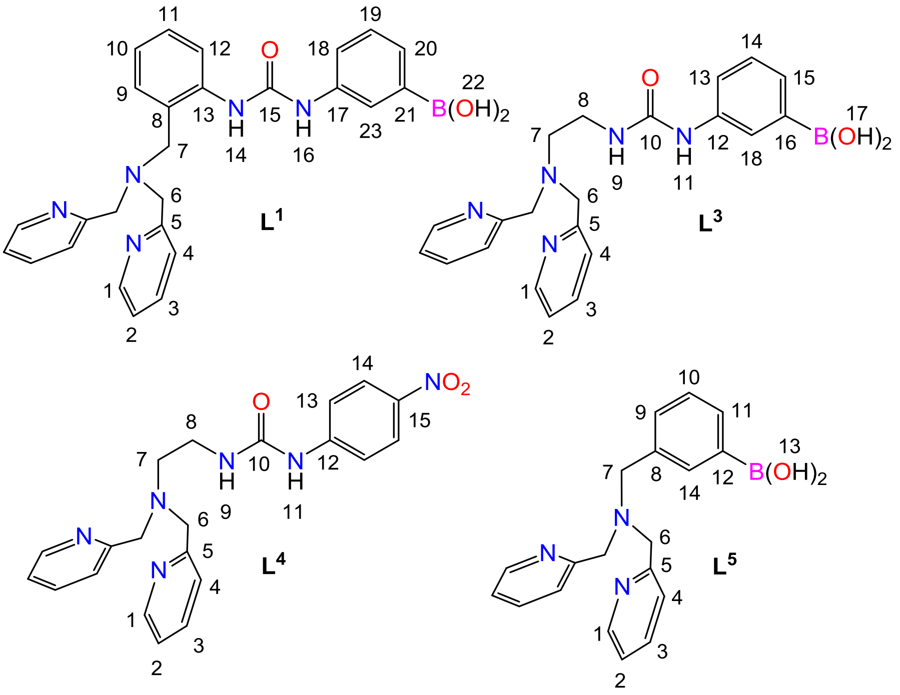

Figure S14. Labelling scheme of ligands L¹, L³, L⁴ and L⁵ for ¹H and ¹³C NMR signals assignment.

[{ZnL[4]}$_2$(μ-PPi)], TPSSh/TZVP, dmso (IEFPM), 0 imaginary frequencies

C	5.79970800	2.99503600	1.63761200
H	6.59373200	2.25348100	1.60132100
C	6.08866100	4.35228900	1.57131300
H	7.11790700	4.69871800	1.47901500
C	5.05216900	5.29362400	1.62462600
C	3.71408800	4.88659400	1.74306600
H	2.92546900	5.63767500	1.78007700
C	3.42089600	3.53440200	1.80804700
H	2.38865800	3.18865700	1.89674200
C	4.45470400	2.55893100	1.75895000
C	4.86982900	0.10982000	1.76414300
C	4.83144500	-2.33714300	1.66348300
H	4.67686600	-2.93237100	2.58090600
H	5.90825400	-2.12847700	1.59923700
C	4.36578000	-3.21606000	0.49671000
H	5.06173700	-4.07491100	0.44191800
H	3.36659500	-3.62612900	0.71209400
C	5.36876800	-1.62197800	-1.14322100
H	6.24187000	-2.14587500	-1.57202800
H	5.72583800	-1.13646200	-0.22228500
C	4.92686200	-0.52108900	-2.08777400
C	5.86078100	0.21033700	-2.83218000
H	6.91927200	-0.05075300	-2.77990300
C	5.41901400	1.26606500	-3.62974600
H	6.13291500	1.84980200	-4.21457700
C	4.05168700	1.55955800	-3.67658600
H	3.66428200	2.37228900	-4.29234400
C	3.17968100	0.77937200	-2.92332000
H	2.09732700	0.92658000	-2.92858900
C	4.14169300	-3.55830300	-1.89314600
H	4.93483700	-4.32366200	-1.82038900
H	4.27308200	-3.04430100	-2.86089700
C	2.77808200	-4.20887500	-1.90680600
C	2.57126600	-5.54825800	-2.24776900
H	3.42290100	-6.19884100	-2.45338400
C	1.25990700	-6.02602600	-2.32453100
H	1.06986400	-7.06800600	-2.59045900
C	0.19803100	-5.15655400	-2.05680100
H	-0.83827900	-5.49367100	-2.10875200
C	0.48501900	-3.83576100	-1.71168800
H	-0.29568400	-3.10139700	-1.49016100
C	-5.79740200	2.99763500	-1.63780100
H	-6.59197000	2.25666800	-1.60132300
C	-6.08529200	4.35509200	-1.57108300
H	-7.11423100	4.70228400	-1.47825800

C	-5.04811100	5.29565200	-1.62467500
C	-3.71039300	4.88763200	-1.74381900
H	-2.92122500	5.63812300	-1.78103500
C	-3.41825400	3.53523200	-1.80920100
H	-2.38632200	3.18870200	-1.89840000
C	-4.45277800	2.56053400	-1.75981200
C	-4.86973600	0.11173600	-1.76494500
C	-4.83308200	-2.33522100	-1.66336100
H	-4.67884300	-2.93096300	-2.58050400
H	-5.90975500	-2.12578600	-1.59927900
C	-4.36811000	-3.21392700	-0.49615000
H	-5.06469600	-4.07224400	-0.44095800
H	-3.36921700	-3.62484200	-0.71128000
C	-5.37006600	-1.61843300	1.14298200
H	-6.24372200	-2.14160600	1.57155400
H	-5.72643400	-1.13272000	0.22188000
C	-4.92759700	-0.51782900	2.08759700
C	-5.86114200	0.21419400	2.83187300
H	-6.91979200	-0.04620600	2.77941800
C	-5.41881000	1.26961900	3.62953000
H	-6.13242200	1.85380800	4.21426300
C	-4.05130100	1.56221700	3.67657300
H	-3.66345200	2.37468300	4.29240200
C	-3.17968800	0.78149300	2.92340700
H	-2.09724400	0.92803500	2.92873500
C	-4.14414600	-3.55507900	1.89389200
H	-4.93761600	-4.32013200	1.82147700
H	-4.27541400	-3.04050900	2.86135700
C	-2.78083600	-4.20626400	1.90806500
C	-2.57474900	-5.54564100	2.24948000
H	-3.42675500	-6.19573300	2.45511000
C	-1.26365400	-6.02405900	2.32665900
H	-1.07420100	-7.06605400	2.59294600
C	-0.20129100	-5.15521600	2.05885000
H	0.83483900	-5.49283700	2.11108500
C	-0.48753400	-3.83438600	1.71326700
H	0.29359600	-3.10053900	1.49154800
N	5.36578200	6.70874800	1.55666800
N	4.27283900	-2.54741800	-0.82537200
N	3.61457900	-0.23156300	-2.14581300
N	1.74922500	-3.38755700	-1.63798900
N	-5.36063400	6.71100000	-1.55630300
N	-4.27473100	-2.54470400	0.82558300
N	-3.61511900	-0.22916500	2.14582000
N	-1.75150000	-3.38552800	1.63921900
O	4.43665600	7.51681300	1.60623700
O	6.54735100	7.04301200	1.45248300

O	6.10526900	0.14708000	1.70494100
O	1.53050700	-1.21665000	0.76288200
O	1.38226100	0.96307800	2.14061900
O	-0.61853500	-0.66496300	2.07414700
O	0.00034100	0.71563200	-0.00094400
O	-1.38160300	0.96254400	-2.14259300
O	-1.53172800	-1.21582500	-0.76301900
O	0.61780500	-0.66716100	-2.07494000
O	-6.54190400	7.04613100	-1.45151200
O	-4.43092400	7.51838200	-1.60613700
O	-6.10514800	0.14991300	-1.70568800
P	-0.60718100	-0.07038000	-1.33661200
P	0.60702000	-0.06985000	1.33547000
N	4.06173000	1.23832100	1.82966000
N	4.16027900	-1.05765100	1.76277400
N	-4.06082700	1.23963800	-1.83090600
N	-4.16102300	-1.05623200	-1.76317800
H	3.02449600	1.08710300	1.93952000
H	3.13693200	-1.04301300	1.62934300
H	-3.02372600	1.08766100	-1.94104800
H	-3.13768200	-1.04221500	-1.62951400
Zn	-2.22381000	-1.40436500	1.09543100
Zn	2.22275100	-1.40614900	-1.09532000

E(RTPSSh) = -7500.964699 Hartree

Zero-point correction= 0.872976
Thermal correction to Energy= 0.937227
Thermal correction to Enthalpy= 0.938171
Thermal correction to Gibbs Free Energy= 0.767868
Sum of electronic and zero-point Energies= -7500.091723
Sum of electronic and thermal Energies= -7500.027473
Sum of electronic and thermal Enthalpies= -7500.026528
Sum of electronic and thermal Free Energies= -7500.196832

[{ZnL[4]}$_2$(μ-H_2PPi)]$^{2+}$, TPSSh/TZVP, dmso (IEFPM), 0 imaginary frequencies

C	-6.09037200	3.09376600	-1.67552700
H	-6.86004900	2.32658700	-1.67140300
C	-6.42773400	4.44072100	-1.60557300
H	-7.47074200	4.75075500	-1.54582400
C	-5.42222900	5.41402500	-1.61528000
C	-4.06989600	5.05641100	-1.69251800
H	-3.30523500	5.83240300	-1.69455400
C	-3.73063500	3.71359400	-1.76062800
H	-2.67935200	3.42318000	-1.81209300

C	-4.73060100	2.70855300	-1.75639300
C	-5.07033400	0.23241800	-1.74442500
C	-4.96399900	-2.21839100	-1.71454500
H	-4.84056700	-2.76375600	-2.66619700
H	-6.03998700	-2.03826800	-1.58876200
C	-4.42410200	-3.14180600	-0.61820900
H	-5.08920800	-4.02394400	-0.59385600
H	-3.42027500	-3.50563400	-0.88783600
C	-5.42489200	-1.67066200	1.14303500
H	-6.30325200	-2.24344500	1.48755800
H	-5.76042300	-1.08540400	0.27421600
C	-5.00427000	-0.68997500	2.21922900
C	-5.95049300	-0.07881800	3.04926100
H	-7.00358700	-0.35026700	2.96009400
C	-5.52840200	0.87174500	3.97889600
H	-6.25282100	1.36061400	4.63323000
C	-4.16722500	1.18179000	4.06717900
H	-3.79438300	1.91348400	4.78470600
C	-3.28061900	0.52483500	3.22038700
H	-2.20521300	0.70634900	3.25155000
C	-4.18128400	-3.64884600	1.74253400
H	-4.95076300	-4.42327000	1.58455100
H	-4.35603800	-3.21674000	2.74253700
C	-2.80296400	-4.26536900	1.75357200
C	-2.58299900	-5.61833500	2.02299600
H	-3.43028100	-6.29300400	2.15424100
C	-1.26825400	-6.07911600	2.12854100
H	-1.06977500	-7.13151700	2.34067300
C	-0.21409800	-5.17689800	1.95975400
H	0.82602400	-5.49573400	2.03711900
C	-0.51273300	-3.84519600	1.67850800
H	0.27067500	-3.09922100	1.53273700
C	6.09087400	3.09235200	1.67652600
H	6.86046100	2.32508100	1.67299900
C	6.42845100	4.43926500	1.60679700
H	7.47154000	4.74917800	1.54785000
C	5.42305800	5.41269300	1.61572900
C	4.07062100	5.05524900	1.69195100
H	3.30605500	5.83133600	1.69338700
C	3.73115100	3.71247700	1.75982700
H	2.67980000	3.42217100	1.81046900
C	4.73099500	2.70731300	1.75638100
C	5.07043800	0.23116200	1.74440400
C	4.96382200	-2.21963300	1.71412200
H	4.84035500	-2.76520400	2.66565300
H	6.03983300	-2.03962300	1.58837400
C	4.42371000	-3.14261700	0.61755800

H	5.08862000	-4.02489100	0.59281600
H	3.41981300	-3.50631600	0.88710700
C	5.42477000	-1.67112700	-1.14321200
H	6.30290000	-2.24405900	-1.48807500
H	5.76061100	-1.08632900	-0.27420100
C	5.00437700	-0.68987700	-2.21898800
C	5.95079900	-0.07855800	-3.04867800
H	7.00382900	-0.35025800	-2.95951300
C	5.52899800	0.87248300	-3.97794800
H	6.25357500	1.36148800	-4.63200600
C	4.16789000	1.18285600	-4.06620300
H	3.79525900	1.91494400	-4.78343600
C	3.28108000	0.52575100	-3.21975400
H	2.20573400	0.70759500	-3.25086600
C	4.18050800	-3.64865900	-1.74338200
H	4.94984500	-4.42331600	-1.58585100
H	4.35516500	-3.21615700	-2.74323100
C	2.80205100	-4.26485300	-1.75442400
C	2.58175200	-5.61770400	-2.02413400
H	3.42886200	-6.29254300	-2.15559900
C	1.26689000	-6.07815700	-2.12969000
H	1.06815200	-7.13046200	-2.34205500
C	0.21296700	-5.17572900	-1.96061300
H	-0.82723600	-5.49429500	-2.03798800
C	0.51193400	-3.84415900	-1.67906800
H	-0.27131700	-3.09806900	-1.53304100
N	-5.78575600	6.82233100	-1.54277000
N	-4.31529900	-2.55793900	0.74877100
N	-3.69531000	-0.38474600	2.31620400
N	-1.77990300	-3.40866400	1.57403200
N	5.78681100	6.82094900	1.54347400
N	4.31493500	-2.55818300	-0.74918500
N	3.69548700	-0.38433600	-2.31592900
N	1.77920900	-3.40793900	-1.57459400
O	-4.88176600	7.65682400	-1.55306800
O	-6.97912900	7.11264900	-1.47459400
O	-6.29662800	0.23743700	-1.62175200
O	-1.62014500	-1.07798300	-0.75657000
O	-1.34064600	1.00136200	-2.14888400
O	0.63178100	-0.67331800	-2.04489000
O	-0.00035800	0.75670500	0.00036600
O	1.34045700	1.00035900	2.14977400
O	1.62033100	-1.07769500	0.75652600
O	-0.63185900	-0.67461200	2.04479300
O	6.98026600	7.11112200	1.47608700
O	4.88292200	7.65556200	1.55318600
O	6.29675700	0.23609600	1.62195300

P	0.55563500	-0.11076900	1.28785700
P	-0.55591900	-0.11037900	-1.28760000
N	-4.30286800	1.39077000	-1.83780700
N	-4.33086900	-0.91637600	-1.78701800
N	4.30305900	1.38958700	1.83760500
N	4.33084700	-0.91755100	1.78688500
H	-3.29521100	1.26865400	-1.96017300
H	-3.31031400	-0.88096200	-1.75391200
H	3.29535100	1.26760900	1.95964700
H	3.31029600	-0.88199700	1.75349100
Zn	2.36460700	-1.44299300	-1.12139900
Zn	-2.36471200	-1.44351600	1.12126200
H	-0.79643200	1.44542400	-2.82566800
H	0.79487600	1.44910700	2.82235000

E(RTPSSh) = -7501.846443 Hartree

Zero-point correction=	0.896949
Thermal correction to Energy=	0.963044
Thermal correction to Enthalpy=	0.963988
Thermal correction to Gibbs Free Energy=	0.790253
Sum of electronic and zero-point Energies=	-7500.949494
Sum of electronic and thermal Energies=	-7500.883398
Sum of electronic and thermal Enthalpies=	-7500.882454
Sum of electronic and thermal Free Energies=	-7501.056189

[ZnL^4(H_2PPi)], TPSSh/TZVP, dmso (IEFPM), 0 imaginary frequencies

C	-4.61470600	-1.33813800	-0.63316700
H	-4.28513400	-2.37386100	-0.64614100
C	-5.93930700	-1.01306400	-0.36695400
H	-6.67348400	-1.79197000	-0.16150700
C	-6.34463500	0.32781200	-0.36114100
C	-5.42899100	1.35924500	-0.61899500
H	-5.76810700	2.39465600	-0.60762600
C	-4.10726600	1.04008800	-0.88383100
H	-3.37531300	1.82495200	-1.08584800
C	-3.67182300	-0.31266600	-0.89956000
C	-1.67763300	-1.75752000	-1.27844500
C	0.48059000	-2.80625400	-1.71299900
H	0.44423800	-3.09918700	-2.77931200
H	0.04268900	-3.64786500	-1.15667400
C	1.96153800	-2.62636300	-1.39385200
H	2.46375800	-3.55544300	-1.72119700
H	2.37729000	-1.80474800	-1.99817700

C	1.59047000	-3.15270500	1.01541000
H	2.27331300	-3.39649200	1.84458200
H	1.25087900	-4.11706900	0.60569100
C	0.42161600	-2.39651100	1.61781500
C	-0.67209500	-3.06422100	2.17826400
H	-0.73431300	-4.15202900	2.11807500
C	-1.67086200	-2.32069000	2.80934300
H	-2.53386900	-2.82240200	3.25166700
C	-1.55452000	-0.92814100	2.86143900
H	-2.31373700	-0.30971100	3.34165900
C	-0.44092700	-0.33397100	2.27373400
H	-0.29791400	0.74777100	2.27173400
C	3.80213400	-2.53479400	0.16993500
H	4.14540100	-3.47611500	-0.29264000
H	4.03049600	-2.59145900	1.24618000
C	4.57109200	-1.36164200	-0.39634000
C	5.79467300	-1.50103300	-1.05549800
H	6.20699900	-2.49489800	-1.23715200
C	6.46986500	-0.35015100	-1.47143200
H	7.42764800	-0.43189200	-1.98938600
C	5.90113900	0.90213400	-1.22271500
H	6.39648000	1.82269300	-1.53430300
C	4.67105200	0.96210000	-0.56805000
H	4.17424100	1.91375700	-0.35343700
N	-7.73194000	0.65581700	-0.08469100
N	2.33990100	-2.35774400	0.02607000
N	0.52037600	-1.05631000	1.67103400
N	4.03218300	-0.15050500	-0.16648700
O	2.74713000	3.58929400	0.17516000
O	1.44802800	3.92772400	2.34831800
O	2.21221500	1.54326000	1.76066600
O	0.36383200	2.68557500	0.39707400
O	-1.00984400	1.89522700	-1.65840100
O	1.21163400	0.71980900	-1.00557600
O	1.22623200	3.12906600	-1.95884400
O	-8.51813200	-0.26573300	0.13937500
O	-8.06287300	1.84250000	-0.08642600
O	-2.24427800	-2.85010600	-1.20669500
P	0.39099900	2.03163600	-1.13286300
P	1.82485500	2.90444100	1.16259500
N	-2.33495300	-0.53165400	-1.16960100
N	-0.33188800	-1.63260700	-1.46916600
H	-1.78450600	0.33126100	-1.35336700
H	0.12020300	-0.70972400	-1.50017300
Zn	2.08031900	-0.14012400	0.60777900
H	1.11683900	3.46503800	3.13800400
H	1.95278900	3.45427600	-1.34510600

E(RTPSSh) = -4356.139077 Hartree

Zero-point correction= 0.473633
Thermal correction to Energy= 0.510868
Thermal correction to Enthalpy= 0.511812
Thermal correction to Gibbs Free Energy= 0.402670
Sum of electronic and zero-point Energies= -4355.665444
Sum of electronic and thermal Energies= -4355.628209
Sum of electronic and thermal Enthalpies= -4355.627265
Sum of electronic and thermal Free Energies= -4355.736408

$[ZnL^4(PPi)]^{2-}$, TPSSh/TZVP, dmso (IEFPM), 0 imaginary frequencies

C	-4.52148600	-1.31819500	-0.67077900
H	-4.21025900	-2.35682700	-0.75160700
C	-5.83593100	-0.98530500	-0.37359700
H	-6.58433900	-1.76101100	-0.21057700
C	-6.21881900	0.36139400	-0.28126200
C	-5.28151100	1.38974800	-0.48410400
H	-5.60200600	2.42845800	-0.40509800
C	-3.96977800	1.06443900	-0.77895400
H	-3.21321100	1.83577600	-0.94364900
C	-3.55502600	-0.29621700	-0.88410300
C	-1.59449300	-1.73747300	-1.35632900
C	0.56965200	-2.76233900	-1.80469500
H	0.58538200	-3.01790600	-2.88240800
H	0.11756000	-3.63016400	-1.30057600
C	2.03296800	-2.57593800	-1.40912300
H	2.56888000	-3.48558200	-1.74404100
H	2.45486300	-1.72258900	-1.96291600
C	1.53020300	-3.15898800	0.95726400
H	2.17561900	-3.44838000	1.80225700
H	1.18965900	-4.10360300	0.50217500
C	0.35108900	-2.41029100	1.55159500
C	-0.74998000	-3.08467000	2.09259300
H	-0.81592500	-4.17168400	2.01617600
C	-1.75037300	-2.34609700	2.72744100
H	-2.61965000	-2.85086700	3.15469400
C	-1.62652500	-0.95475800	2.80583600
H	-2.38624900	-0.34276400	3.29432800
C	-0.50563000	-0.35580900	2.23395600
H	-0.32740900	0.72223500	2.25286200
C	3.77293600	-2.49050400	0.26264400

H	4.16932700	-3.41602900	-0.19480000
H	3.93480300	-2.56739700	1.34985400
C	4.56313500	-1.29518200	-0.22390200
C	5.82790400	-1.40975000	-0.80814100
H	6.25733800	-2.39576100	-0.99556100
C	6.52121300	-0.24207800	-1.14048500
H	7.51109900	-0.30222500	-1.59858600
C	5.92958200	0.99782000	-0.88356500
H	6.44300300	1.92919200	-1.12935100
C	4.65662000	1.03638400	-0.30860300
H	4.12677300	1.97935700	-0.07997900
N	-7.59170900	0.69538700	0.02481400
N	2.32581000	-2.34238300	0.03108100
N	0.45228200	-1.07491600	1.62421000
N	4.00366000	-0.09665400	0.00847700
O	2.97249100	3.48367300	0.52430400
O	1.09885700	3.88026300	2.30551200
O	1.92878300	1.49503900	1.80750500
O	0.49087700	2.76503200	0.10154500
O	-0.99317300	1.74909600	-1.67517800
O	1.24791500	0.63516700	-1.15193300
O	1.21173300	2.89912300	-2.41252900
O	-8.39658700	-0.22315800	0.20515600
O	-7.90587300	1.88720300	0.09347300
O	-2.17134100	-2.83312800	-1.33889500
P	0.50292900	2.03702700	-1.38708000
P	1.72521100	2.97583900	1.25659300
N	-2.23250800	-0.51017800	-1.17722800
N	-0.25513000	-1.60280600	-1.55377600
H	-1.65498400	0.38732300	-1.35193900
H	0.21623600	-0.66923900	-1.50298700
Zn	1.95268600	-0.00586900	0.56407300

E(RTPSSh) = -4355.161522 Hartree

Zero-point correction= 0.448855
Thermal correction to Energy= 0.485014
Thermal correction to Enthalpy= 0.485958
Thermal correction to Gibbs Free Energy= 0.379022
Sum of electronic and zero-point Energies= -4354.712667
Sum of electronic and thermal Energies= -4354.676509
Sum of electronic and thermal Enthalpies= -4354.675565
Sum of electronic and thermal Free Energies= -4354.782501
